# Tumor-associated macrophage derived IL-6 enriches cancer stem cell population and promotes breast tumor progression via Stat-3 pathway

**DOI:** 10.1186/s12935-022-02527-9

**Published:** 2022-03-17

**Authors:** N. N. V. Radharani, Amit S. Yadav, Ramakrishna Nimma, T. V. Santosh Kumar, Anuradha Bulbule, Venkatesh Chanukuppa, Dhiraj Kumar, Srinivas Patnaik, Srikanth Rapole, Gopal C. Kundu

**Affiliations:** 1 Laboratory of Tumor Biology, Angiogenesis and Nanomedicine Research, National Centre for Cell Science (NCCS), Pune, 411007 India; 2 School of Biotechnology, KIIT Deemed To Be University, Bhubaneswar, 751 024 India; 3 Kalinga Institute of Medical Sciences (KIMS), KIIT Deemed To Be University, Bhubaneswar, 751024 India; 4 Present Address: Department of Cancer Biology, The University of Texas MD Anderson Cancer Center, Houston, TX 77054 USA; 5 Proteomics Laboratory, National Centre for Cell Science (NCCS), Pune, 411007 India

**Keywords:** Tumor-associated macrophages, Cancer stem cells, IL-6, Breast cancer progression

## Abstract

**Background:**

Cancer stem cells (CSCs) play crucial role in tumor progression, drug resistance and relapse in various cancers. CSC niche is comprised of various stromal cell types including Tumor-associated macrophages (TAMs). Extrinsic ques derived from these cells help in maintenance of CSC phenotype. TAMs have versatile roles in tumor progression however their function in enrichment of CSC is poorly explored.

**Methods:**

Mouse macrophages (RAW264.7) cells were activated by interaction with conditioned media (CM) of murine breast cancer cells (4T1) into TAMs and the effect of activated macrophage (TAM) derived factors was examined on enrichment of cancer stem cells (CSCs) and tumor growth using in vitro and in vivo models.

**Results:**

In this study, we report that macrophages upon interaction with breast cancer cells activate tumor promoting function and exhibit differential expression of various proteins as shown by secretome analysis using proteomics studies. Based on secretome data, we found that Interleukin-6 (IL-6) is one of the up-regulated genes expressed in activated macrophages. Further, we confirm that TAMs produce high levels of IL-6 and breast cancer cell derived factors induce IL-6 production in activated macrophages via p38-MAPK pathway. Furthermore, we demonstrate that tumor activated macrophages induce enrichment of CSCs and expression of CSC specific transcription factors such as Sox-2, Oct-3/4 and Nanog in breast cancer cells. We further prove that TAM derived IL-6 plays a key role in TAM mediated CSC enrichment through activation of Signal transducer and activator of transcription 3 (STAT-3) signaling. TAM derived IL-6 influences breast cancer cell migration and angiogenesis. Moreover, our in vivo findings indicated that TAM derived IL-6 induces CSC population and resulting tumor growth in breast cancer.

**Conclusion:**

These finding provide evidence that TAM derived IL-6 plays a major role in CSC enrichment and tumor progression in breast cancer and IL-6 and its regulated signalling network may act as potential therapeutic target for management of breast cancer.

**Supplementary Information:**

The online version contains supplementary material available at 10.1186/s12935-022-02527-9.

## Introduction

Breast cancer in one of the top most commonly diagnosed cancer among women worldwide [1]. Breast tumor progression is a very complex multi-stage process in which interaction between cancer and stromal cells occur via regulation of various cytokines, chemokines, and components of the extracellular matrix which play a crucial role in promoting tumor growth, metastasis and angiogenesis [2]. Within microenvironment, along with cancer cells there are various stromal cells like cancer associated fibroblasts, endothelial cells, immune cells like macrophages, neutrophils, NK cells etc. those also play critical role in tumor tissue remodelling, promoting cancer cell proliferation and angiogenesis which further aids in tumor progression [3].

Accumulated evidences indicated that immune cells within tumor microenvironment play an important role in tumor progression [2, 3]. Macrophages are one of the most common populations among inflammatory cells within breast tumor micro-environment which have been known to be involved in promoting major hallmarks of cancer. Within tumor micro-environment, cytokines released by tumor cells suppress the immune function of macrophages and polarise them into M2 phenotype which promote tumor growth, metastasis and angiogenesis and termed as tumor-associated macrophages (TAMs) [4–6]. Recent studies have revealed that TAMs exhibit distinct sub populations depending upon their location in tumor micro-environment, stage, and type of tumor. Hence, polarized state of tumor promoting TAMs is still ambiguous and under discussion and depending upon the type, number and various signal from outside or within tumor micro-environment, TAMs act as double-edged sword and partially decides the development of tumor and angiogenesis [7, 8].

IL-6 a is a pleotropic cytokine known for its versatile role in diverse functions such as inflammation, immune response, haematopoiesis, proliferation of non-immune cells, cellular metabolism and inducing synthesis of acute phase proteins. Studies have indicated that IL-6 plays dual role both in promoting tumor growth and inducing adaptive immunity within the tumor microenvironment [9–11]. Interestingly, it has been shown that IL-6 can be secreted both by cancer and stromal cells like cancer associated fibroblasts (CAFs), TAMs, and endothelial cells in various cancers and induces proliferation, angiogenesis and metastasis by activating STAT-3 pathway [9]. Moreover, few reports have suggested that interaction of cancer cells with macrophages induces IL-6 expression, however the mechanism by which cancer cells regulate IL-6 expression in TAMs is not well defined [12].

Cancer stem cells (CSCs) are small population of cells within tumor which possess the ability of self-renewal and differentiation and have role in tumor initiation, progression and expansion due to intrinsic alterations in the tumor microenvironment. Various reports have demonstrated CSCs role in breast tumor growth, metastasis and resistance to therapy [13]. Interestingly, studies have observed co-localization of TAMs with CSCs and identified the role of TAMs in regulation of CSCs via secretion of various cytokines [14, 15]. However, the soluble mediators secreted by TAMs that are involved in regulation of CSCs have not been explored comprehensively yet.

In the present study, we have delineated the signalling mechanism through which breast cancer cells induce IL-6 production in TAMs. We further demonstrate the role of TAMs derived IL-6 in enrichment of CSCs and tumor progression through STAT-3 pathway in breast cancer using both in vitro and in vivo models.

## Materials and methods

### Maintenance of cell lines

Mouse mammary carcinoma (4T1-luc2-tdTomato) (Caliper Life Sciences, USA), murine macrophages (RAW264.7) (ATCC, USA) were cultured and maintained in Dulbecco's Modified Eagle Medium (DMEM, Gibco, Thermo Fisher Scientific, USA) along with 10% fetal bovine serum (Gibco, Thermo Fisher Scientific, USA), 100 units penicillin and 100 μg/ml streptomycin (Himedia, India). Human umbilical vein endothelial cells (HUVEC) (ATCC, USA) were maintained in Endothelial Cell Growth Medium as per manufacturer’s instructions (Lonza, USA).

### Activation of macrophages and preparation of conditioned media (CM)

4T1 cells were seeded and CM were collected by adding serum free media for 24 h. RAW264.7 macrophages were activated into TAMs by treating them with CM of 4T1 for 12 h. The media of activated macrophages (TAMs) was replaced with incomplete media for another 24 h to collect the CM of TAMs. After 24 h, media were collected, centrifuged, and supernatants were collected for further experimentations.

### Secretomic analysis

The cytokine profile of RAW264.7 macrophages in response to CM of 4T1 was analysed by quantitative secretomics analysis using label free conditions. Detailed methods are described in Additional file 1: Section.

### Estimation of IL-6 expression

Since secretomic analysis demonstrated 11-fold increase in IL-6 in activated macrophages, the amount of IL-6 secreted from the CM of RAW264.7 cells was estimated using IL-6 ELISA Kit (BioLegend, San Diego, CA, USA). Briefly, RAW264.7 cells (5 × 10^5^) were seeded and then treated with CM of 4T1 cells in 1:1 ratio for 12 h. After treatments, supernatants were collected and the levels of IL-6 were quantified by ELISA according to manufacturer's instructions. In a separate experiments, IL-6 expression in CM of 4T1 cells was analysed by ELISA.

### Isolation of TAMs from 4T1 breast tumors

To isolate TAMs from tumors, 4T1 tumors developed in BALB/c mice were collected and processed for sorting using FACS with anti-F4/80 antibody. Detailed protocol is shown in Additional file 1: Section.

### Mammosphere formation assay

4T1 cells were either grown alone or co-cultured with CM of activated macrophages for 12 h. After treatments, cells were trypsinzed, counted and seeded in low attachment plates at a density of 1 × 10^4^ cells/well. Cells were allowed to grow in serum free DMEM and nutrient mixture F-12 medium supplemented with B27 (1:50), 20 ng/ml IGF, 20 ng/ml EGF, and 20 ng/ml FGF for 4–5 days and observed for sphere formation. Mammospheres formation was then analysed by counting the number of spheres per high-power field (hpf) and plotted as fold increase in number of mammopsheres vs control.

### Western blot

The western blot was conducted as described earlier [16]. Briefly, the expression of p-p38, c-Jun, c-Fos, actin in RAW264.7 cells and Sox-2, Oct ¾, Nanog, p-STAT3, STAT3 and actin in 4T1 cells were analyzed by western blot. Details of antibodies are provided in Additional file 1: Table S1.

### Immunofluorescence

The immunofluorescence was performed as described earlier [17]. To examine the role of CM of 4T1 cells in inducing IL-6 expression, RAW264.7 cells were treated with CM of 4T1 for 24 h followed by treatment with Brefaldin for 4 h and IL-6 expression was examined by immunofluorescence. In separate experiments, RAW264.7 cells were treated with CM of 4T1 for 24 h and levels of p-p38, c-Fos and c-Jun were examined by immunofluorescence. Briefly, cells were fixed with paraformaldehyde, quenched using 0.1% glycine then permeabilized by 0.1% TritonX-100, blocked with 10% FBS, stained for respective proteins using their specific antibodies followed by their secondary antibodies, mounted with DAPI and images were taken by Confocal microscopy (Leica) and analysed using LAS AF LITE software.

To examine the role of activated macrophages in inducing CSC mediated breast tumor progression, 4T1 cells were treated with either CM of activated macrophages or IL-6 neutralized CM of activated macrophages and immunofluorescence experiments were performed. In separate experiments, 4T1 cells were treated with recombinant IL-6 and immunofluorescence studies were conducted. Briefly, cells were fixed, quenched and then stained for Sox-2, Oct-3/4, Nanog and p-STAT using their specific antibodies followed by respective secondary antibodies and analyzed by confocal microscopy (Leica) and quantified using LAS AF LITE software.

### Chromatin immunoprecipitation (ChIP) assay

To examine the binding of AP-1 (c-Jun:c-Fos) with IL-6 promoter in response to CM of 4T1 treatment, the putative binding site in IL-6 promoter was analyzed as described under the Additional file 1: Section. Sequences of primers used in the assay are provided in Additional file 1: Table S2.

### Transwell migration assay

The cell migration was examined by performing Transwell migration assay by using Transwell cell culture chambers (Corning, NY, USA) as described previously [18].

### In vitro tube formation assay

HUVECs were maintained in EGM-2 medium (Lonza) as per the manufacturer’s instructions. Tube formation assay was carried out as reported previously [19]. Briefly, 96-well plates were coated with growth factor depleted Matrigel and HUVEC (1 × 10^4^ cells/well) were added. Cells were treated with either recombinant IL-6 (50 ng/ml) or CM of activated RAW264.7 cells or IL-6 neutralized CM of activated RAW264.7 cells. In another set of experiments, CM of activated RAW264.7 cells pre-treated with SB203580 were used. Photographs were taken after 6 h using Inverted microscope (Nikon). Number of tube junctions were analysed by using AngioTool64 0.6a software and described as fold change as compared to control in the form of bar graph.

In a separate experiments, 4T1 cells were treated with either CM of activated RAW264.7 cells or IL-6 neutralized CM of activated RAW 264.7 cells. In another set, 4T1 cells were pre-treated with Stattic followed by treatment with CM of activated RAW264.7 cells. The conditioned media were acquired and used for in vitro tube formation assay using HUVECs as described above.

### In vivo tumorigenicity

All animal procedures were conducted as per the Guidelines approved by ‘Committee for the Purpose of Control and Supervision of Experiments on Animals’ (CPCSEA), Government of India with prior approval from Institutional Animal Care and Use Committee (IACUC) of National Centre for Cell Science (NCCS), Pune, India. Briefly, 4T1 breast cancer cells (5 × 10^3^) were injected in the right mammary fat pad of 4–6-week-old female BALB/c mice. After development of tumor (1.5–2 mm), CM of activated RAW264.7 cells (200 µl) or IL-6 neutralized CM of activated RAW264.7 cells (200 µl) were intra-tumorally injected every alternate day for two weeks. Tumor dimensions were measured using verniar caliper and tumor volume was calculated using following equation,

$$ {\text{Tumor volume }} =\uppi /{6}\left( {{\text{d1 }} \times {\text{ d2}}} \right){3}/{2;} $$

where d1 is the length and d2 is the width of the tumor. After two weeks, mice were euthanized by CO_2_ asphyxiation, tumors were removed, imaged and weighed.

### Statistical analysis

The results of ELISA, q-PCR, wound migration, tube formation, and tumorigenicity assays are expressed as mean ± SE or ± SD. Statistical significance were analysed by Student’s ‘t’ test using Sigma Plot 10.0 software. p value of < 0.05 was considered as significant.

## Results

### Breast cancer conditioned media (CM) activate macrophages and enhances IL-6 expression

**Fig. 1:**
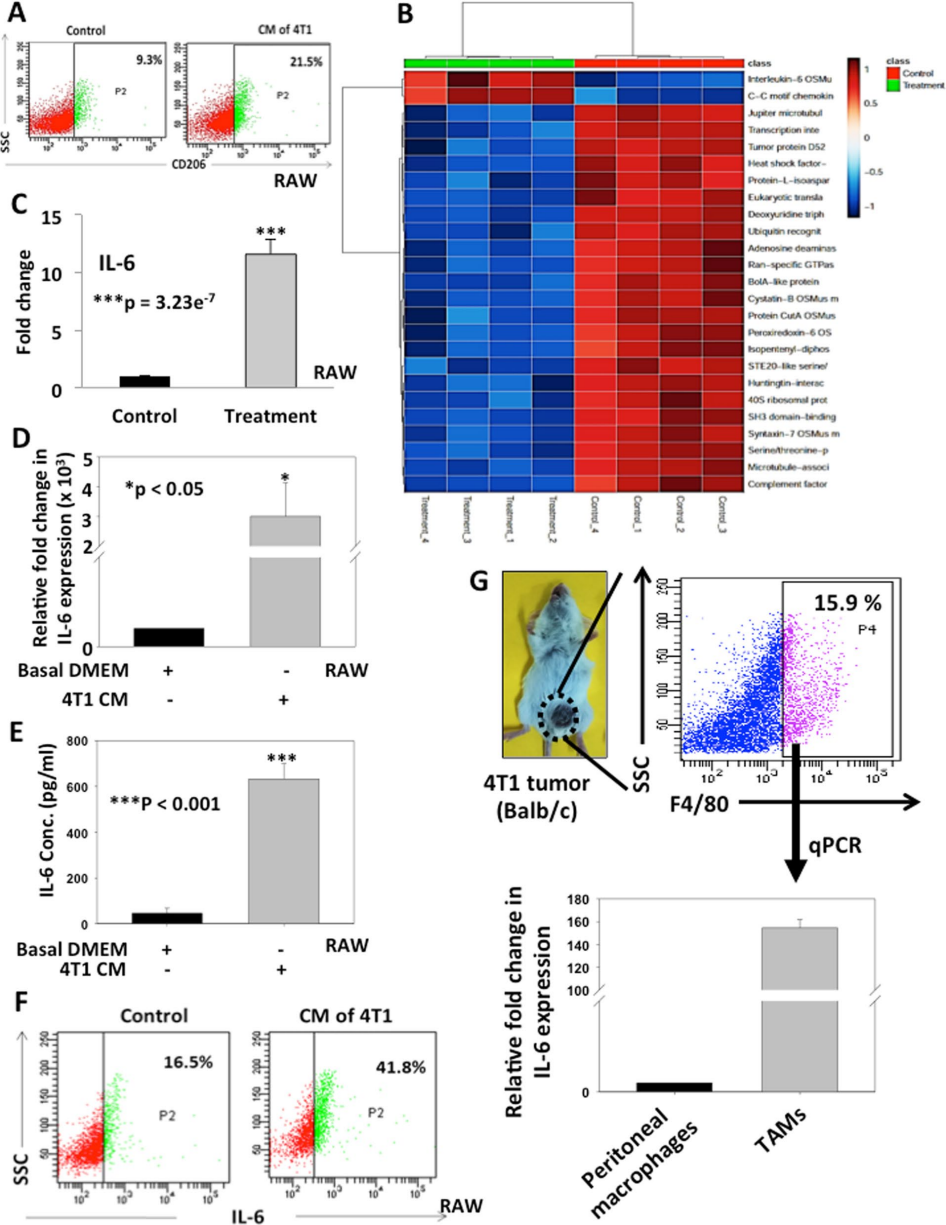
Breast Cancer Conditioned Media (CM) activate macrophages and enhances IL-6 expression. **A** Macrophages (RAW264.7 cells) were treated with CM of 4T1 cells for 24 h and expression of CD206 was examined by BD FACSCanto II analyser. **B** RAW264.7 cells were either cultured alone or treated with CM of 4T1 for 12 h and CM were collected and analysed for secretomics by Orbitrap Fusion mass spectrometer (ThermoScientific™, USA). Heatmap showing the differentially expressed secretory proteins in control and CM of activated macrophage **C** Bar graph showing fold increase in IL-6 expression in CM of activated macrophages compared to control. Error bars represent mean ± SEM, ***denotes p < 0.001, n = 3 independent experiments. **D** Bar graph represents the relative fold change in IL-6 expression in activated RAW264.7 cells compared to control by qRT-PCR analysis and calculated using ΔΔCt method. β-actin served as endogenous control for normalization. Error bars represent mean ± SEM, *denotes p < 0.05, n = 4 independent experiments. **E** Bar graph represents IL-6 concentration in CM of activated RAW264.7 cells vs control as examined by ELISA. Error bars represent mean ± SEM, ***denotes p < 0.001, n = 4 independent experiments. **F** RAW264.7 cells were treated with CM of 4T1 for 24 h. Brefeldin A was added to inhibit the secretion of IL-6. After treatment, cells were processed and analysed for IL-6 expression using FACS Canto II analyser. **G** Bar graph represents the relative fold change in IL-6 expression by q-PCR analysis in TAMs isolated from 4T1 breast tumors using F4/80 marker by FACS sorting. Peritoneal macrophages were used as control. β-actin served as endogenous control

To elucidate the role of cancer cells in activation of macrophages into TAMs, we co-cultured macrophages (RAW264.7) with CM of breast cancer cells (4T1) and expression of CD206 was analyzed as TAM marker. The results indicated that co-cultured macrophages acquired a change in morphology compared to control (Additional file 1: Fig. S1). Further an increase in CD206 expression (21.5%) was observed in co-cultured macrophages as compared to control (9.3%) indicating activation of macrophages into TAMs (Fig. 1A). To understand whether increased expression of TAMs specific marker is also associated with differential expression profiles of secretory proteins in activated macrophages, we performed secretome analysis using CM of activated RAW264.7 cells using Orbitrap Fusion Mass Spectrometry and analysed by Proteome Discoverer software. The data showed differential expression of secretory proteins including cytokines in activated macrophages with nearly 117 upregulated proteins. Interestingly, IL-6 was one of the highly upregulated proteins with 11-fold increase (Fig. 1B, C). Thus, secretomics data provided potential candidate proteins regulated by breast cancer cells in TAMs. We further corroborated our findings in clinical settings by correlating the expression of the TAMs specific genes and IL-6 in breast cancer clinical specimens using TCGA datasets. We correlated the genomic levels of TAM specific marker CD163 with IL-6 in TCGA database using cBioPortal platform. The data from three different datasets used in this study showed that increased CD163 expression was correlated with enhanced IL-6 expression with Pearson correlation coefficients of 0.35. 0.46 and 0.44 for Pan-Cancer Atlas, METABRIC and The Metastatic Breast Cancer Project (MBC) datasets respectively (Additional file 1: Fig. S2). Overall, the data suggest that TAMs may be associated with IL-6 production in clinical settings of breast cancer.

To further validate increased expression of IL-6 in activated macrophages as found in our secretomics data, we co-cultured RAW264.7 cells with CM of 4T1 cells under in vitro conditions and the expression of IL-6 was analysed by q-PCR and sandwich ELISA. The data suggested a significant increase of IL-6 level at the transcriptional as well as at the protein level in activated macrophages compared to control (p < 0.05 and p < 0.001 respectively) (Fig. 1D and E). Our FACS data also exhibited enhanced expression of IL-6 (41.8%) in activated macrophages in comparison to control (16.5%) which was further supported by immunofluorescence findings (Fig. 1F and Additional file 1: Fig. S3A, B). We also found similar result when peritoneal macrophages isolated from mouse were treated with CM of 4T1 where IL-6 was found to be enhanced in activated peritoneal macrophages as compared to control (Additional file 1: Fig. S3C). Thus, our data suggests that breast cancer cells induce IL-6 expression in activated macrophages. Additionally, to confirm our in vitro findings under in vivo conditions, we have generated 4T1 tumors in mammary fat pads of female BALB/c mice and TAMs were isolated from 4T1 tumors using F4/80 murine macrophage specific marker by FACS. IL-6 expression was analysed in isolated TAMs by qPCR and compared with peritoneal macrophages isolated from same mouse. The results exhibited higher expression of IL-6 in TAMs as compared to control peritoneal macrophages (Fig. 1G). Hence, the data depicts that TAMs express high levels of IL-6 in breast tumors.

### Breast cancer cells induce p-38 mediated AP-1 dependent IL-6 expression in tumor activated macrophages

**Fig. 2:**
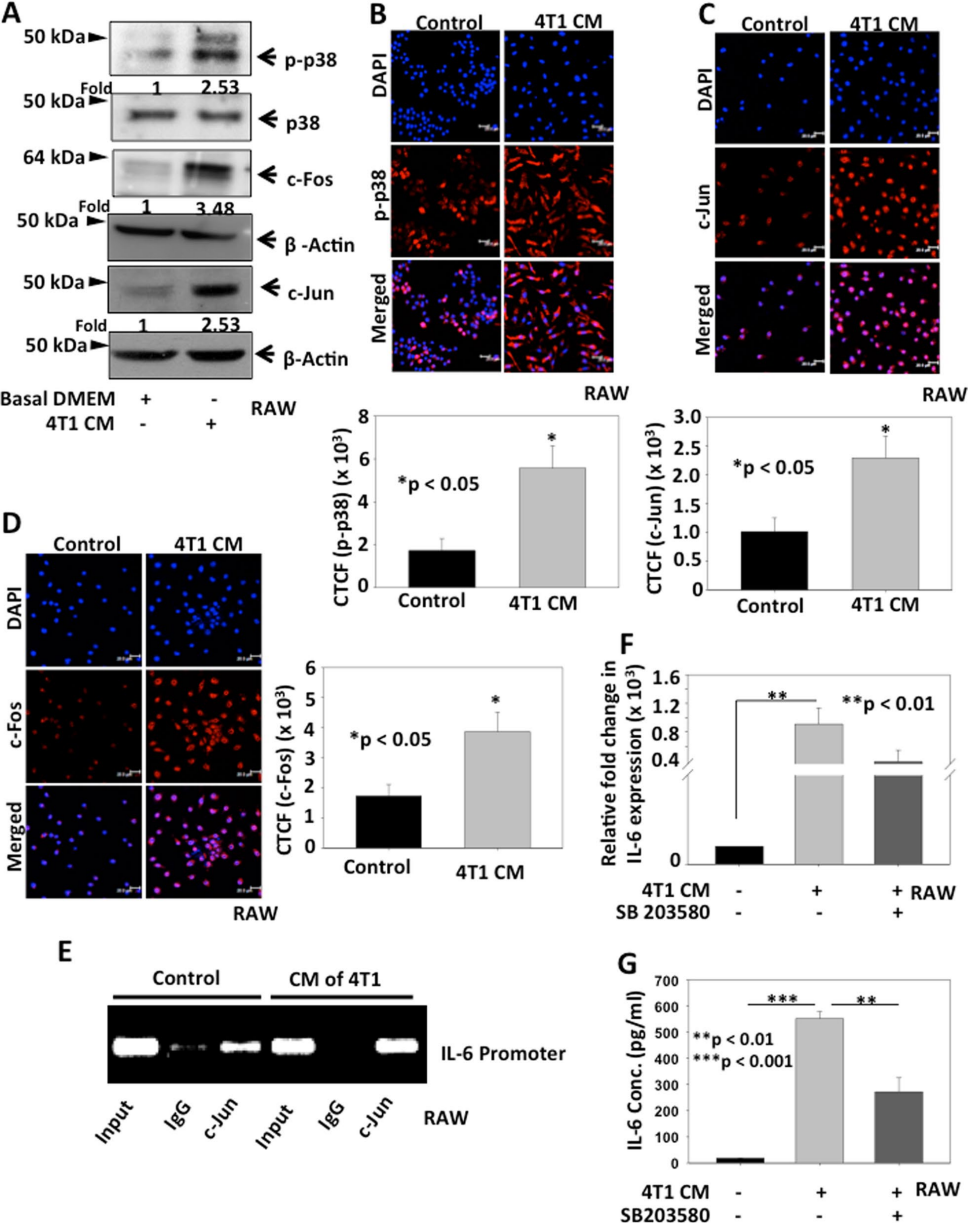
Breast cancer cells induce p-38 mediated AP-1 dependent IL-6 expression in tumor activated macrophages. **A** RAW264.7 cells were treated with CM of 4T1 cells and expressions of p-p38, c-Fos and c-Jun were analysed by western blot. Actin was used as loading control. Densitometry analysis was performed using NIH-ImageJ software. Relative fold changes with respect to control are shown. **B-D** RAW264.7 cells were treated with CM of 4T1 cells and expressions of p-p38, c-Jun and c-Fos were analysed by immunofluorescence study. Bar graphs represent corrected total cell fluorescence (CTCF) of p-p38, c-Jun and c-Fos respectively as quantified by ImageJ software, mean ± SEM, *denoted p < 0.05, n = 3 independent experiments). **E** Binding of AP-1 to IL-6 promoter upon CM of 4T1 treatment. RAW264.7 cells were treated with CM derived from 4T1 cells and ChIP analysis was performed by immunoprecipitation with c-Jun antibody and PCR amplified using AP-1 specific primers. **F** Bar graph represents the relative fold change in IL-6 expression in RAW264.7 cells treated with 4T1 CM with or without pre-treatment with SB203580 compared to control by qRT-PCR analysis and calculated using ΔΔCt method. β-actin served as control for normalization, mean ± SEM, **denotes p < 0.01, n = 5 independent experiments. **G** Bar graph represents IL-6 concentration in CM of RAW264.7 cells treated with 4T1 CM with or without pre-treatment with SB203580 by ELISA, graph represents 1 of 3 technical repeats

Studies have indicated the role of p-38 pathway in inducing IL-6 expression in macrophages [6]. Thus, in order to delineate the signalling mechanism involved in breast cancer cell mediated expression of IL-6 in activated macrophages, we checked the effect of CM of breast cancer cells on phosphorylation of p38 in macrophages. RAW264.7 cells were treated with CM of 4T1 cells and phosphorylation of p38 was analysed by western blotting. The data suggests that macrophages co-cultured with CM of breast cancer cells shown enhanced phosphorylation of p-p38 (2.53-fold) as compared to macrophages cultured alone (Fig. 2A). Various studies have demonstrated the role of Activating Protein-1 (AP-1), a dimeric complex composed of c-Fos and c-Jun, as a downstream regulator of gene expression in p38-MAPK pathway [20]. AP-1 binds to the consensus AP-1 binding site of various genes and regulates their expression. Interestingly, AP-1 has been reported to have binding site in promoter region of IL-6. Therefore, to delineate the role of AP-1 in inducing IL-6 expression in activated macrophages, the levels of c-Fos and c-Jun in activated RAW264.7 cells were analysed by western blot. The data indicated that both c-Fos and c-Jun levels were increased (3.58 and 2.53-fold respectively) in CM of 4T1 treated RAW264.7 macrophages as compared to control (Fig. 2A). The immunofluorescence data also displayed similar results suggesting that breast cancer cells activate p38 signaling pathway in activated macrophages (Fig. 2B–D). To further confirm the mechanism of transcriptional activation of IL-6 in RAW264.7 cells in response to CM of 4T1 treatment, ChIP assay was performed. Our in-silico analysis identified putative binding site for AP-1 transcription factor on IL-6 promoter. ChIP data using c-Jun antibody exhibited that CM of 4T1 significantly enhanced binding of AP-1 on IL-6 promoter in RAW264.7 cells suggesting that breast cancer cell-derived CM can promote transcriptional activation of IL-6 in macrophages by inducing p38 mediated AP-1 binding on IL-6 promoter (Fig. 2E). Further, to investigate the role of p-38 signaling in enhancing IL-6 expression in activated macrophages, RAW264.7 cells were pre-treated with p38 inhibitor SB203580, followed by treatment with CM of 4T1 cells and expression of IL-6 was analysed by q-PCR and ELISA. The data revealed an increased expression of IL-6 in activated macrophages, whereas IL-6 expression got abrogated when RAW264.7 cells pre-treated with p38 inhibitor (Fig. 2F, G). FACS data also supported these results where enhanced IL-6 expression (39%) was restricted by SB203580 treatment (26.7%) in CM of 4T1 treated macrophages as compared to control (17.1%) (Additional file 1: Fig. S3D). Overall, these findings suggested that breast cancer cells induce IL-6 expression in tumor activated macrophages via p-38 signaling.

### TAMs enhance stemness in breast cancer cells

**Fig. 3:**
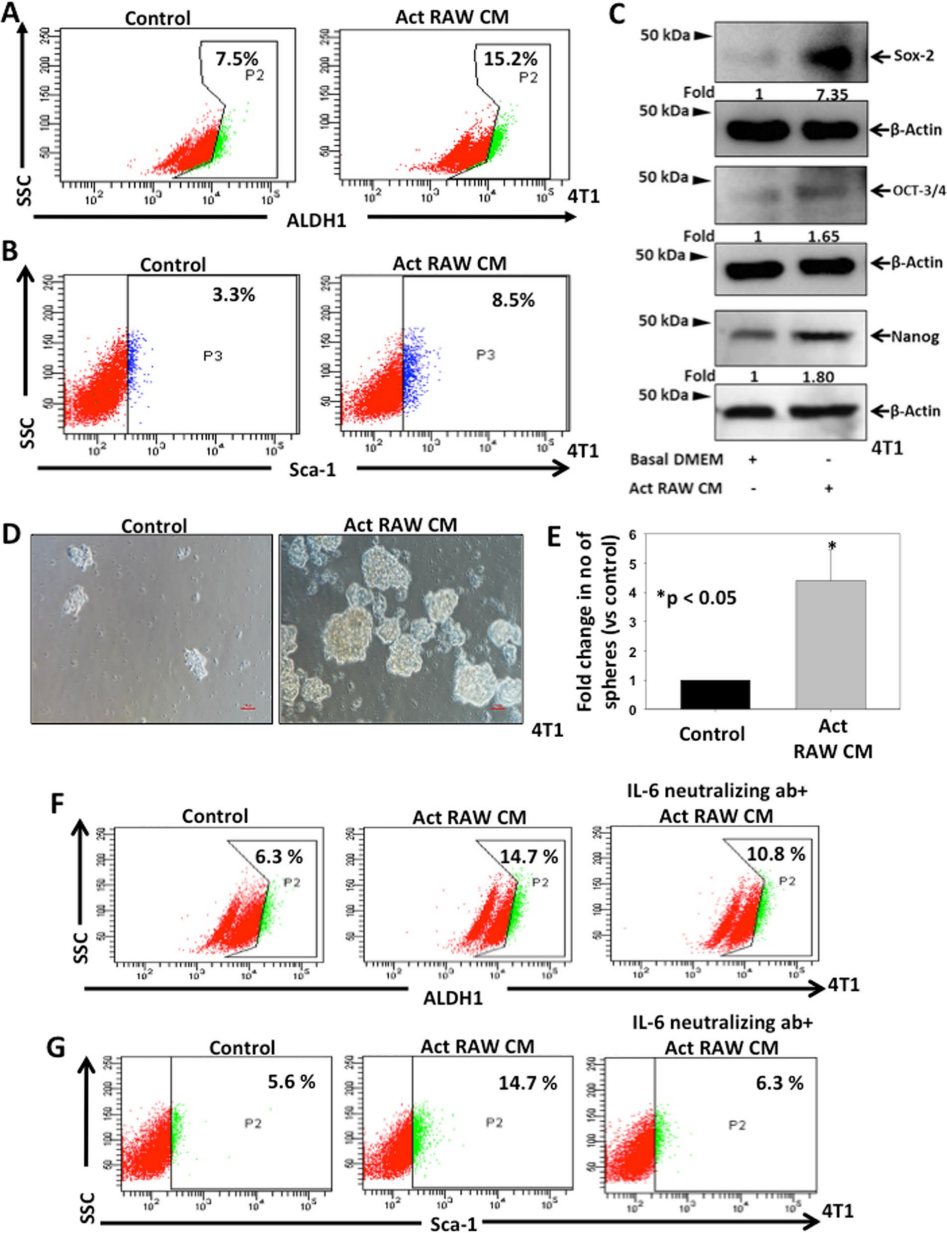
TAM derived IL-6 enriches CSCs in breast cancer. **A**, **B** 4T1 cells were treated with CM of activated RAW264.7 cells for 24 h and ALDH1 activity and expression of Sca-1 were examined by FACSCanto II analyser. **C** 4T1 cells were treated with CM of activated RAW264.7 cells for 24 h and expressions of Sox-2, Oct 3/4 and Nanog were analysed by western blot using their specific antibodies. Actin was used as loading control. Densitometry analysis was performed using NIH-ImageJ software. Relative fold changes with respect to control are shown. **D** 4T1 cells were treated with CM of activated RAW264.7 cells and seeded in low attachment plates for mammosphere formation. **E** Bar graph represents fold change in number of mammospheres vs control**,** mean ± SEM, *denotes p < 0.05, n = 3 independent experiments. **F**, **G** 4T1 cells were treated with either CM of activated RAW or CM of activated RAW neutralized with IL-6 antibody (20 µg/ml) and ALDH1 activity as well as Sca1 expression were examined by flow cytometry

Since CSCs play an important role in enhancing tumor progression, we investigated the role of TAMs in enrichment of CSCs in breast cancer. 4T1 cells were co-cultured with CM of activated RAW264.7 cells for 24 h and CSCs enrichment was analysed by flow cytometry using Sca-1 expression and ALDH1 activity by aldefluor assay as CSC specific markers. Our data revealed that 4T1 cells treated with CM of activated RAW264.7 cells exhibited higher ALDH1 activity (15.2%) and Sca-1 expression (8.5%) when compared to control cells (7.5% and 3.3% respectively) (Fig. 3A, B). Moreover, when 4T1 cells were co-cultured with RAW264.7 cells, higher expression of Sca-1 (5.5%) was observed in co-cultured 4T1 cells compared to 4T1 cells cultured alone (2.8%) (Additional file 1: Fig. S4A). Thus, our data suggested that tumor activated macrophages enhance CSCs phenotype in breast cancer. We next examined the role of activated macrophages in regulation of CSC phenotype by examining the expression of CSC specific transcriptional factors Sox-2, Oct3/4 and Nanog in breast cancer cells by western blotting, q-PCR and immunofluorescence. Western blot analysis demonstrated significant increase in the expression of Sox-2 (7.35-fold), Oct3/4 (1.65-fold) and Nanog (1.80-fold) in CM of activated RAW treated 4T1 cells as compared to control (Fig. 3C). Similar results were found in our q-PCR and immunofluorescence analysis (Additional file 1: Fig S4B–G).

Further to validate the role of activated macrophages in promotion of stem cell properties in breast cancer cells, we performed mammosphere assay with 4T1 cells treated with CM of activated RAW264.7 cell. Our results demonstrated that higher number of mammospheres formed (4.4-fold) in 4T1 cells treated with CM of activated macrophages (Fig. 3D, E). Overall, the data depicted that tumor activated macrophages have the ability to induce CSC enrichment in breast cancer.

### TAM derived IL-6 enriches CSCs in breast cancer

Since IL-6 was found to be one of the highly upregulated cytokines in activated macrophages, we sought to explore the role of activated macrophage derived IL-6 in enrichment of breast CSCs. Prior to examine the role of TAM derived IL-6 in breast CSC enrichment, we first examined the levels of IL-6 in CM of 4T1 cells by ELISA to determine the possible autocrine effect leading to CSC enrichment. The data suggested that 4T1 cells secrete very low amount of IL-6 which was around six-fold lower than IL-6 secreted by activated RAW (Additional file 1: Fig S5). To delineate the role of TAM derived IL-6 on breast CSC enrichment, 4T1 cells were treated with either CM of activated RAW264.7 or CM of activated RAW264.7 neutralized with IL-6 antibody and ALDH1 activity as well as Sca-1 expression were examined by flow cytometry. The data indicated that 4T1 cells treated with CM of activated RAW264.7 cells exhibit higher ALDH1 activity (14.7%) and Sca-1 expression (14.7%) when compared to respective controls (6.3% and 5.6% respectively). Furthermore, this effect was supressed when IL-6 was neutralized in the CM (10.8% and 6.3% respectively) (Fig. 3F, G). We further examined the status of dual positive (ALDH1^+^ Sca-1^+^) population in identical conditions and found similar results where treatment with activated RAW264.7 CM enhanced dual positive population (12.4%) with respect to control (3.3%) and the effect is abrogated when IL-6 was neutralized (8.1%) (Additional file 1: Fig S6). Therefore, our findings suggest that tumor activated macrophage derived IL-6 regulates CSC phenotype in breast cancer cells.

### IL-6 alone is sufficient to enhance CSC phenotype in breast cancer cells

Since TAM derived IL-6 was found to have a role in breast CSC enrichment, it prompted us to examine whether IL-6 alone can enhance CSCs enrichment in breast cancer cells. 4T1 cells were treated with recombinant mouse IL-6 (50 ng/ml) for 24 h and CSC phenotype was examined by analysing Sca-1 expression and ALDH1 activity using flow cytometry. The results exihibited that IL-6 treatment upregulated Sca-1 expression (15.9%) and ALDH1 activity (13.4%) as compared to their respective controls (7.2% and 7.3%) in 4T1 cells (Additional file 1: Fig S7 A, B). Further, our immunoblotting and immunoflurescence data showed that CSC specific transcription factors, Sox-2, Oct 3/4 and Nanog were enhanced in cancer cells upon treatment with IL-6 (Additional file 1: Fig. S7 C–F). Thus, our data indicate that IL-6 alone can enhance breast cancer stemness by regulating CSC phenotype and stem cell specific transcription factors expression in breast cancer.

### TAM derived IL-6 enhance stemness in breast cancer through STAT-3 pathway

**Fig. 4:**
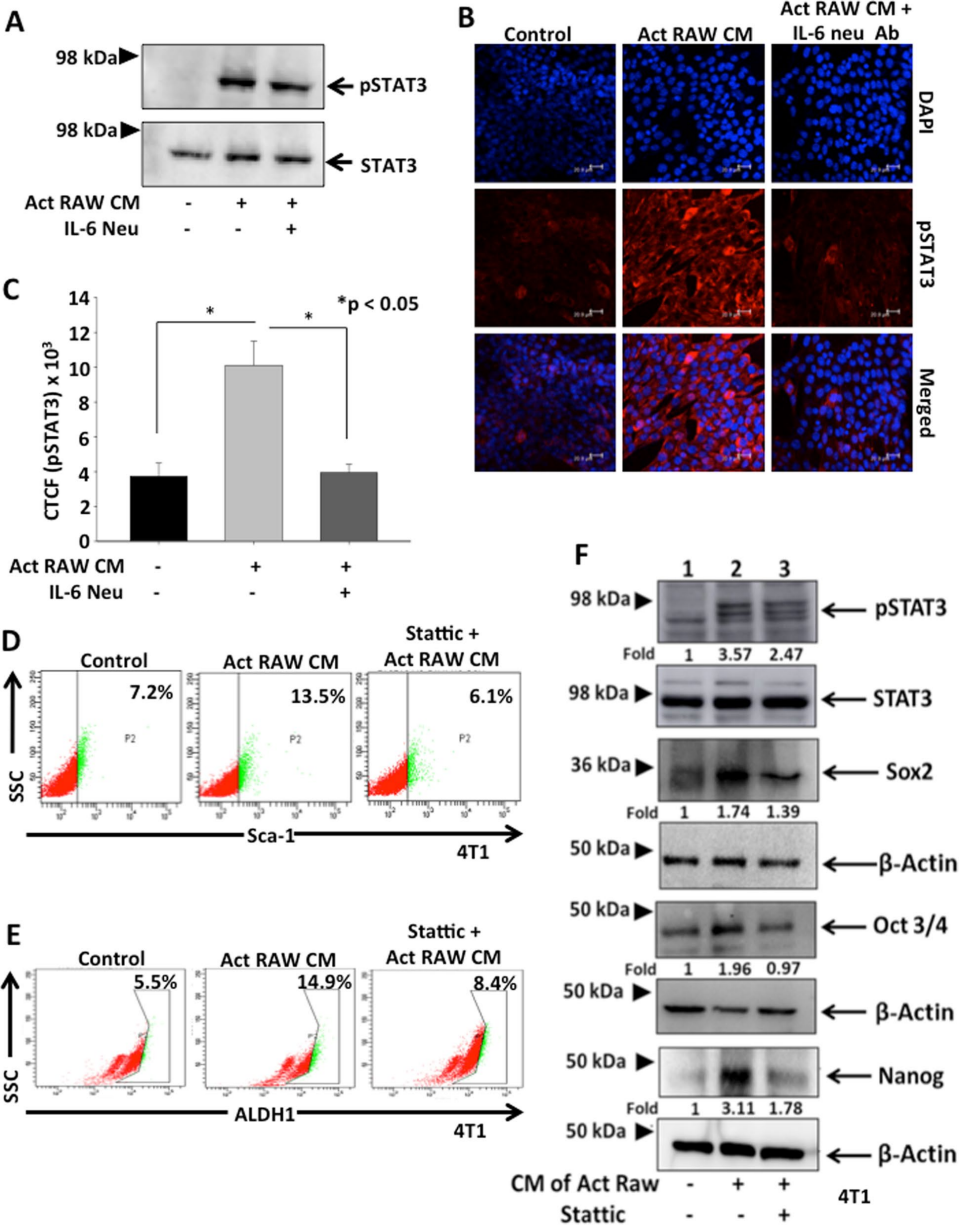
TAM derived IL-6 enhance stemness in breast cancer through STAT- 3 pathway. **A** 4T1 cells were treated with either CM of activated RAW264.7 cells or CM of activated RAW264.7 cells neutralized with IL-6 antibody (20 µg/ml) and expression of p-STAT-3 was examined by western blotting. STAT-3 was used as loading control. **B**, **C** In similar conditions, expression of p-STAT-3 was examined by immunofluorescence in 4T1 cells. Bar graphs represent corrected total cell fluorescence (CTCF) of p-STAT-3 as quantified by ImageJ software, mean ± SEM, *denotes p < 0.05, n = 3 independent experiments. **D**, **E** 4T1 cells were either untreated or treated with Stattic (5 µM), a STAT-3 inhibitor, and then treated with CM of activated RAW264.7 and CSC phenotype was examined by analysing Sca-1 expression and ALDH1 activity using flow cytometry. **F** 4T1 cells were either untreated or treated with Stattic (5 µM), a STAT-3 inhibitor, and then treated with CM of activated RAW264.7 and expression of p-STAT-3, Sox-2, Oct-3/4 and Nanog were analysed by western blotting. STAT-3 and Actin were used as respective loading control. Densitometry analysis was performed using NIH-ImageJ software. Relative fold changes with respect to control are shown

Previous reports have demonstrated that IL-6 exerts its action via the signal transducers gp (glycoprotein) 130 receptor which leads to the activation of the JAK/STAT (Janus kinase/signal transducer and activator of transcription) cascade [21]. STAT-3 is one of the members of STAT family which is known to be activated in different types of cancer. Therefore, we examined the role of TAM derived IL-6 on phosphorylation of STAT-3 in 4T1 cells by western blot and immunofluorescence. The data revealed that 4T1 cells treated with CM of activated RAW264.7 cell enhanced phosphorylation of STAT-3, however the effect was abrogated when IL-6 was neutralized in CM of activated macrophages (Fig. 4A–C).

Further, in order to confirm the role of STAT3 signaling in TAM derived IL-6 mediated CSC enrichment in breast cancer, 4T1 cells were pre-treated with Stattic, a specific inhibitor of STAT3, followed by treatment with CM of activated RAW264.7 cells or recombinant IL-6 (50 ng/ml) and Sca-1 expression and ALDH1 activity was examined by flow cytometry. The data revealed that treatment with CM of activated RAW264.7 enhanced Sca-1 expression (13.5%) and ALDH1 activity (14.9%) in cancer cells as compared to their respective controls (7.2 and 5.5% respectively) and this effect was inhibited on pre-treatment with Stattic (6.1 and 8.4% respectively) (Fig. 4D, E). Similar results were observed when 4T1 cells were treated with recombinant IL-6 in presence or absence of Stattic pre-treatment (Additional file 1: Fig S8 A, B). We next evaluated phosphorylation of STAT-3 in cancer cells on treatment with CM of activated RAW264.7 cells by western blotting and our results revealed that STAT-3 phosphorylation was elevated (3.57-fold) in 4T1 cells and the effect was abrogated on pre-treatment with Stattic (2.47-fold). Moreover, CM of activated RAW264.7 cells upregulated the expression of stem cell specific transcription factors Sox-2, Oct-3/4 and Nanog which were supressed by pre-treatment with Stattic (Fig. 4F). Taken together, these findings demonstrate that TAM derived IL-6 enhances the CSC phenotype in breast cancer cells through STAT-3 pathway.

### TAM derived IL-6 augments cell migration and angiogenesis in breast cancer

**Fig. 5:**
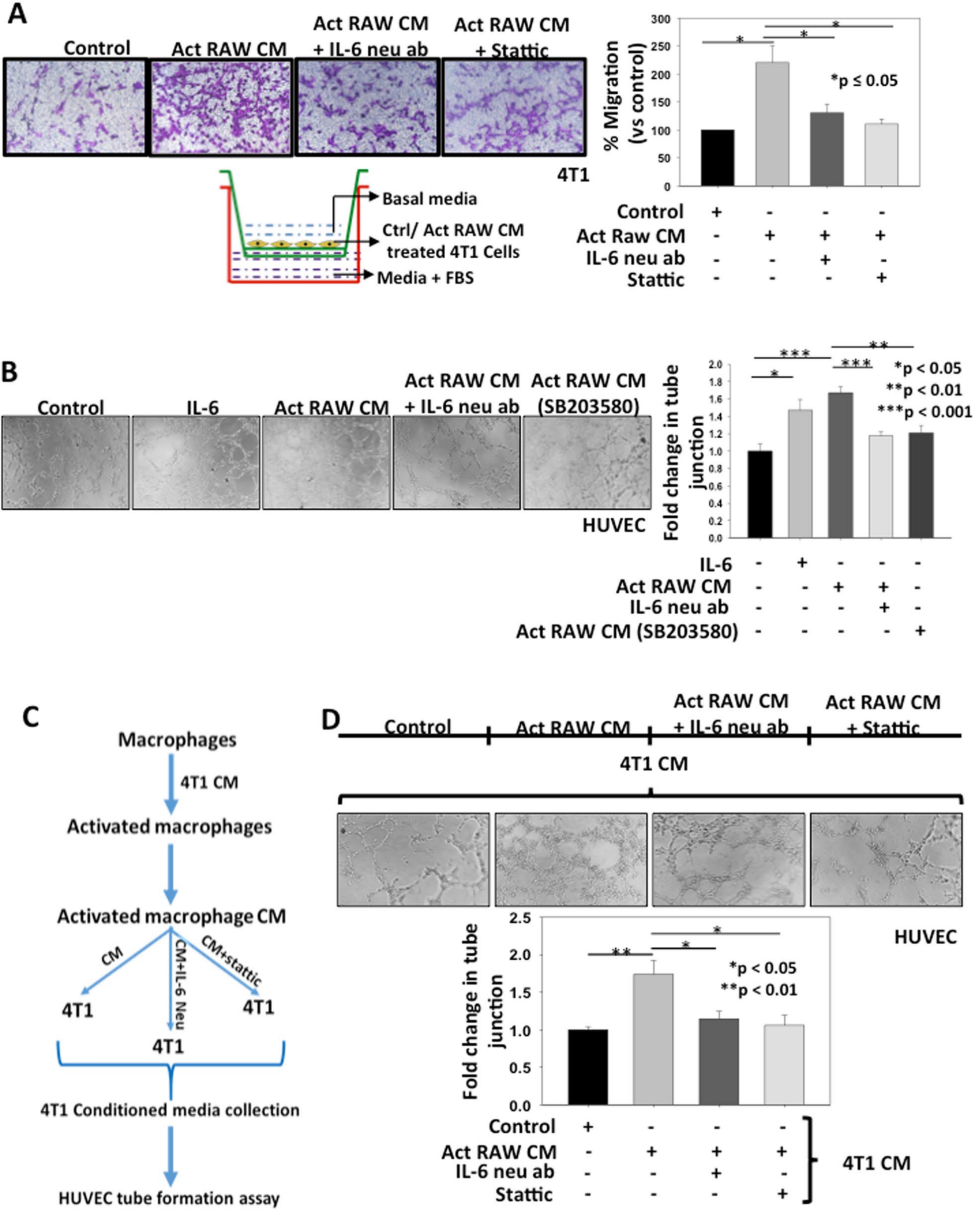
TAM derived IL-6 augments migration and angiogenesis in breast cancer. **A** 4T1 cells were treated with CM of activated RAW264.7 cells or CM neutralized with IL-6 antibody (20 µg/ml). In separate experiments, 4T1 cells were pre-treated with Stattic (5 µM) followed by treatment with CM of activated RAW264.7. Cells were seeded in the upper chamber. In all groups, fetal bovine serum (10%) containing media was used in the lower chamber as chemoattractant. After 18 h, migrated cells were stained and quantified statistically using NIH-ImageJ software and displayed in the form of bar graph. The error bar represents mean ± SEM, *denotes p < 0.05, n = 3 independent experiments. **B** RAW264.7 cells were either treated with CM of 4T1 cells or pre-treated with SB203580 (10 µM) followed by treatment with CM of 4T1 cells and CM were collected after 12 h. In separate experiments, IL-6 was neutralized in CM of activated RAW264.7 cells by using IL-6 neutralizing antibody (20 µg/ml) and used for tube formation assay using HUVECs. In another setting, HUVECs were treated with recombinant IL-6 and tube formation assay was performed. After treatment, tubular structure was photographed and analysed. Number of junctions were measured by using AngioTool64 0.6a software and described as fold change compared to control in the form of bar graph. Error bars represent mean ± SEM, *denotes p < 0.05, **denotes p < 0.01 and ***denotes p < 0.001. **C**, **D** 4T1 cells were treated with either CM of activated RAW264.7 cells or CM neutralized with IL-6 antibody (20 µg/ml). In separate experiments, 4T1 cells were pre-treated with Stattic (5 µM) followed by treatment with CM of activated RAW264.7 cells. The media were replaced with fresh basal DMEM for 12 h and CM of 4T1 cells were collected and used for in vitro tube formation assay using HUVECs. After treatment, tubular structure was photographed and analysed. Number of junctions were measured using AngioTool64 0.6a software and expressed as fold change compared to control in the form of bar graph. Error bars represent mean ± SEM, *denotes p < 0.05, **denotes p < 0.01

To explore the role of TAMs derived IL-6 in inducing metastasis in cancer cells, we performed transwell migration assay. 4T1 cells were treated with CM of activated RAW264.7 cells or IL-6 neutralized CM of activated RAW264.7 CM. In separate experiments, 4T1 cells were pre-treated with Stattic followed by treatment with CM of activated RAW264.7 cells and transwell migration assay was performed. The data suggested that treatment with CM of activated macrophages enhanced migration of 4T1 cells by 2.2-fold as compared to control which was significantly suppressed by IL-6 neutralization (1.3-fold) or Stattic treatment (1.1-fold) indicating that TAM derived IL-6 promotes breast cancer cell migration via STAT-3 pathway (Fig. 5A). In separate experiments, wound assay was performed using 4T1 cells in identical treatment conditions. The data reproduce similar results as observed in transwell migration assay where IL-6 neutralization or static treatment abrogated the CM of Act RAW264.7 mediated 4T1 cell migration (Additional file 1: Fig. S9). These findings indicate that TAM derived IL-6 enhances cell migration by activating STAT3 pathway in breast cancer.

To examine whether TAM derived IL-6 can promote angiogenesis in breast cancer, we performed HUVECs tube formation assay. RAW264.7 cells were either treated with CM of 4T1 cells or pre-treated with SB203580 then with CM of 4T1 cells and conditioned media were collected. In separate experiments, IL-6 was neutralized in CM of activated RAW264.7 by using IL-6 neutralizing antibody and used for tube formation assay. These data demonstrated that HUVECs treated with either recombinant IL-6 or CM of activated macrophages showed higher tube formation and increased number of junction’s (1.5 and 1.7-fold respectively) thereby inducing angiogenesis. This effect was abrogated when IL-6 was neutralized in CM of activated macrophages (1.17-fold) or activated macrophages were treated with SB203580 prior to CM collection (1.2-fold) (Fig. 5B). Thus, our results depicted that TAM derived IL-6 have the ability to induce angiogenesis and IL-6 alone is sufficient to induce angiogenesis in breast cancer.

To further elucidate the role of activated macrophages in promotion of angiogenic potential of breast cancer cells, 4T1 cells were treated with either CM of activated RAW264.7 or CM of activated RAW264.7 neutralized with IL-6 antibody. In a separate experiments, 4T1 cells were pre-treated with Stattic followed by treatment with CM of activated RAW264.7. The media were replaced with fresh basal DMEM for 12 h and conditioned media were acquired and used for in vitro tube formation assay using HUVECs. The data suggests that treatment with CM of activated macrophage significantly induced the breast cancer cell mediated angiogenesis (1.7-fold increase in tube junction vs control) and the effect was attenuated upon IL-6 neutralization (1.2-fold) or Stattic treatment (1.1-fold) (Fig. 5C, D). These data indicate that TAM derived IL-6 enhances angiogenic potential of breast cancer cells through STAT-3 pathway.

### TAMs derived IL-6 promotes tumor growth in breast cancer

**Fig. 6:**
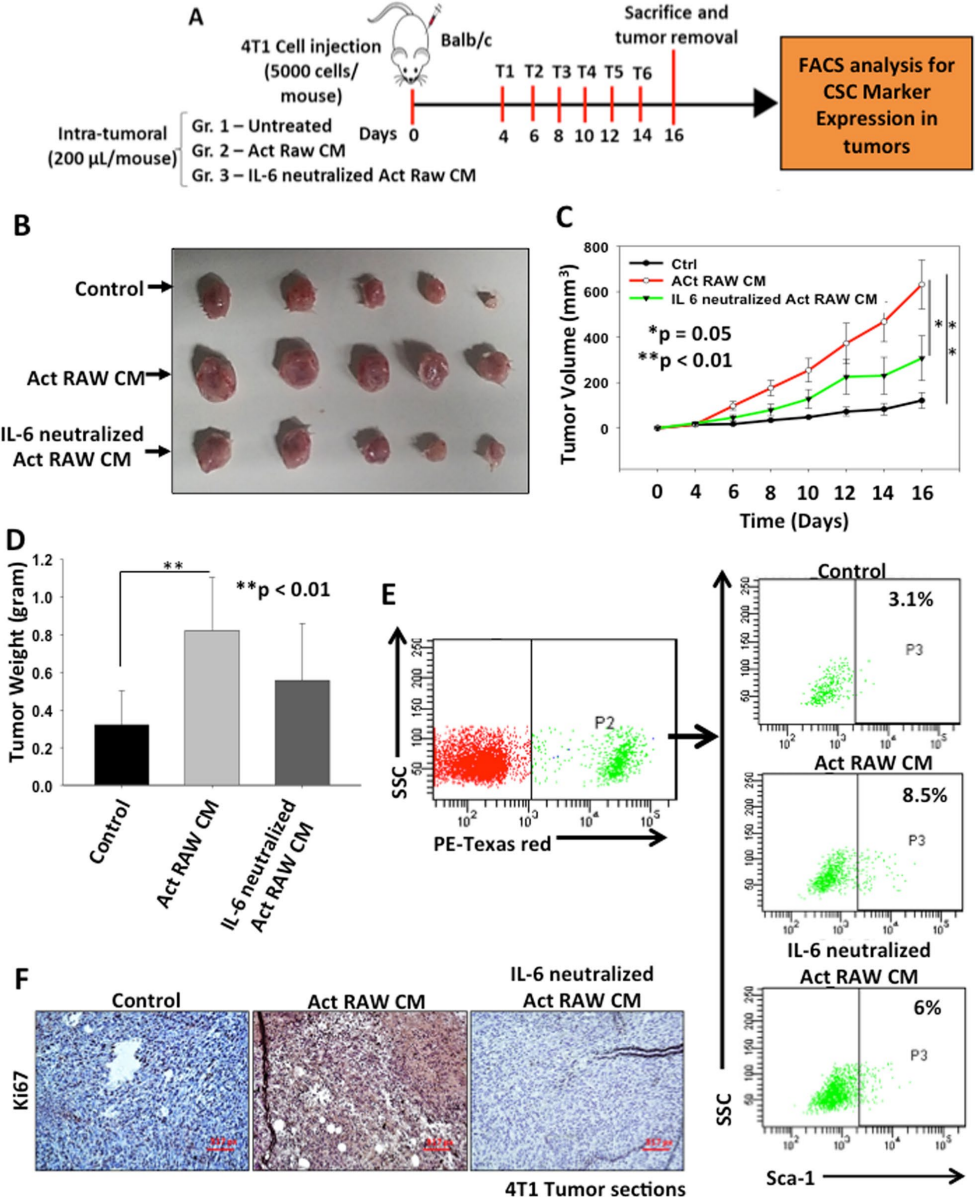
TAMs derived IL-6 promotes tumor growth in breast cancer. **A** Schematic representation of experimental plan. Briefly, 4T1 cells were injected into the mammary fat pad of female BALB/c mice and when the tumor reached palpability, mice were treated with either CM of activated RAW264.7 cells or IL-6 neutralized CM of activated RAW264.7 cells and tumor growth was observed. After treatments, mice were sacrificed, tumors were excised and tumor weight and volume were observed. **B** Digital photographs of excised tumors. **C** Tumor volumes were calculated and analysed statistically. The line graph describes change in mean tumor volume with respect to time in different groups. Data is represented in mean ± SE, *denotes p < 0.05, **denotes p < 0.01, n = 5. **D** Tumors were excised, weighed and analysed statistically. Bar graph represents mean tumor weight ± SD, **denotes p < 0.01, n = 5. **E** FACS analysis was performed to check the CSC population from these tumors. Breast cancer cells population was selected based on their endogenous expression of tdTomato fluorescent protein and expression of Sca-1 was examined in these populations using FACS analysis. **F** Expression of Ki67 in control or Act RAW CM and IL-6 neutralized Act RAW CM treated 4T1 tumor sections examined by immunohistochemistry

To investigate the role of activated macrophages derived IL-6 in enhancing breast tumor growth, orthotopic breast tumors were generated in female BALB/c mice. 4T1 cells were injected into the mammary fat pad of mice and when the tumor reached to palpability, CM of activated RAW264.7 or IL-6 neutralized CM of activated RAW264.7 cells was administrated intra-tumorally and tumor growth was observed. The results revealed that mice treated with CM of activated macrophages exhibit higher tumor growth compared to control, whereas the increase in tumor size was abrogated when the IL-6 is neutralized in CM (Fig. 6A–D and Additional file 1: Fig S10). We further processed the excised tumors through enzymatic digestion, cells were separated and primary cultures were established. 4T1 cell line used to establish tumors was luciferase and red fluorescent protein (tdTomato) expressing cell line. Breast cancer cell population was selected based on their endogenous expression of tdTomato fluorescent protein in PE-Texas red channel in FACS analysis and CSC population was examined. Our results suggest that CM of activated RAW264.7 cells treated tumor possess higher CSCs (8.5%) compared to control (3.1%) and IL-6 neutralized CM treated tumors (6%) (Fig. 6E). Immunohistochemical analysis of tumor sections revealed enhanced expression of Ki67 in CM of Act RAW264.7 treated tumor which was inhibited after neutralization of IL-6 suggesting that TAM derived IL-6 induced cell proliferation in breast cancer (Fig. 6F). Thus, our in vivo findings suggest that, TAM derived IL-6 promotes CSC enrichment and tumor growth in breast cancer.

## Discussion

Interaction of cancer cells with surrounding stroma is a key phenomenon which leads to reprogramming of stromal components to create a tumor microenvironment favourable for tumor progression. Previously it has been demonstrated that cancer cells induce chemokine mediated upregulation of p38-MAPK and ERK pathway in an autocrine manner which leads to tumor progression in melanoma [20]. Recent report has shown that breast cancer cell derived osteopontin induces the resident fibroblast to myofibroblast differentiation which subsequently results in breast tumor progression [22]. Similarly, various other studies have established the role of cross-talk between cancer cell and stromal cells in mutual regulation and resulting tumor progression [22]. In this study, we sought to understand the molecular mechanism of tumor-stromal interaction especially the crosstalk between macrophages and breast cancer cells in activation of macrophages into TAMs and TAM dependent enrichment of CSCs and tumor progression in breast cancer. Our work deciphers the mechanism through which breast cancer cells induce IL-6 expression in tumor activated/educated macrophages. We further demonstrate that TAM derived IL-6 induces CSC enrichment in breast cancer via STAT3 pathway. Moreover, we establish that TAM derived IL-6 promotes metastasis, angiogenesis and tumor growth in breast cancer by in vitro and in vivo studies. Therefore, we first activated macrophages (RAW264.7) by treatment with the CM of breast cancer cells (4T1) to convert them into tumor educated macrophages or tumor-associated macrophages (TAMs). Previously, our group have shown that tumor cell conditioned media can educate macrophages into tumor promoting type [20]. Several studies have shown that TAMs in the tumor micro-environment exhibit CD206 phenotype in different cancers [23, 24]. We observed that macrophages co-cultured with cancer cells exhibit higher CD206 expression indicating that cancer cells derived factor(s) modulated macrophages phenotype into TAMs. Further, to elucidate the mutual effect of crosstalk between macrophages and breast cancer cells, we emphasized on studying soluble factors secreted by these activated macrophages in response to CM of breast cancer cells by secretome analysis. The data revealed differential expression of cytokines and growth factors and remarkably IL-6 was among the top of ten upregulated cytokines in CM of activated macrophages. Recent studies supported our observation and showed that TAMs exhibit high IL-6 expression in various cancers [22, 26]. TCGA analysis also demonstrated positive correlation between expression of TAM marker and IL-6. We further validated our secretomics data by ELISA, qPCR, FACS, immunofluorescence along with in vivo studies showing that TAMs demonstrate higher IL-6 expression at both transcriptional and translational levels as compared to normal macrophages. Most importantly, our data demonstrate that CM of 4T1 cells could induce IL-6 expression in mouse peritoneal macrophage suggesting that breast cancer cell can activate and enhance IL-6 expression in normal macrophages. Overall, these data suggested that breast cancer induces IL-6 expression in TAMs. Various reports have suggested the role of p38-MAPK pathway in regulation of IL-6 in macrophages. Wang et al. have demonstrated that Colistin, an immunostimulatory agent, can enhance IL-6 expression in macrophages by activating p38 MAPK pathway [27]. Further, macrophages have been shown to express IL-6 in response to LPS via p38 MAPK pathway [28]. In light of this, we intended to study the role of breast cancer cells in regulation of p38-MAPK pathway in macrophages and the data suggested that activated macrophages have higher phosphorylation of p38 as compared to control. The p38 pathway imparts its activity by inducing downstream molecules such as c-Jun and c-Fos and form heterodimeric complex AP-1 which can upregulate various downstream genes by binding to their promoter [20]. Interestingly, it has been reported that IL-6 promoter contains AP-1 binding sites suggesting the role of AP-1 in IL-6 expression in macrophages [28]. ChIP analysis affirmed our results where pull down of AP-1 using c-Jun antibody revealed enhanced binding of AP-1 in IL-6 promoter in CM of 4T1 treated RAW264.7 cells. In order to confirm the role of p38 signalling in regulation of IL-6 expression in TAMs, SB203580, a specific inhibitor of p38 MAPK was used. Treatment with SB203580 resulted in abrogation of enhanced IL-6 expression in macrophages in response to CM of breast cancer cells as confirmed by in vitro studies. Thus, our results indicate that soluble factor derived from breast cancer cells can upregulate the expression of IL-6 in TAMs via p38 pathway through AP-1 mediated transcriptional regulation in TAMs.

Previous literature has indicated the role of TAMs in regulation of CSCs and promotion of tumor growth [12, 14, 29, 30]. Thus, we further examined the role of activated macrophages in breast CSC regulation and tumor progression. Our results exhibited that treatment with CM of activated macrophages induces expression of CSC specific markers (ALDH1 activity and Sca-1 expression) in breast cancer cells as compared to control. Further, an increase in expression of stem cell specific transcription factors Sox-2, Oct3/4 and Nanog in response to CM of activated macrophages validated these findings. A plethora of studies have established that CSCs have property of mammospheres formation in suspension cultures due to their inherent anoikis resistant nature [31, 32]. Our data revealed that CM of activated macrophages treated 4T1 cells form higher number of mammosphere than untreated cells hence confirming that activated macrophages induce CSC enrichment in breast cancer.

Accumulated evidences have shown the role of IL-6 in progression of different types of cancers [33–35]. However, the function of IL-6 is paradoxical as it contributes in both pro and anti-tumorigenic activities. Interestingly, few reports have demonstrated the role of IL-6 in enrichment of CSC population leading to cancer progression [12]. Moreover, only few studies have explored the role of TAM derived IL-6 in regulation of CSCs specifically in breast cancer. We have shown here that recombinant IL-6 treatment enhanced CSC specific marker as well as transcription factor expression in breast cancer cells suggesting that IL-6 alone is able to induce CSC phenotype in breast cancer. These results provide a clue that activated macrophage derived IL-6 may be one of the cytokines involved in CSC enrichment in breast cancer. Here, we observed enhanced expression of CSC specific markers, Sca-1 and ALDH1 which was abrogated in breast cancer cells when IL-6 was neutralized in the CM of activated RAW264.7 cells. These findings suggest that indeed TAM derive IL-6 induces CSC enrichment in breast cancer. Various reports have demonstrated that IL-6 regulates its various functions in different types of cancer primarily through JAK/STAT pathway [17, 36–38]. We examined the levels of p-STAT3 expression in response to CM of TAMs and/or recombinant IL-6 in breast cancer cells and our results exhibited enhanced phosphorylation of STAT-3 upon treatment with either recombinant IL-6 or CM of activated macrophages and the effect was abrogated when IL-6 was neutralized in CM of activated RAW264.7 cells suggesting that IL-6 may act through JAK/STAT pathway to regulate CSCs in breast cancer. Further, the role of STAT-3 pathway in IL-6 mediated CSC enrichment in breast cancer was confirmed by using Stattic, a STAT-3 inhibitor, and it was observed that pre-treatment with Stattic attenuated activated macrophage derived IL-6 mediated CSC enrichment in breast cancer. Additionally, cancer cells treated with recombinant IL-6 also showed increased CSCs expression which was abrogated upon pre-treatment with Stattic. Thus, our data demonstrates that TAM derived IL-6 mediates STAT-3 dependent enrichment of CSCs in breast cancer.

Metastasis is one of the hallmarks of cancer and is associated with cancer cell migration, invasion and tumor endothelial interaction [39]. Earlier, Wang et al. have shown that CAF derived IL-6 increases metastatic potential of lung cancer cells via STAT3 pathway [40]. Our results suggested that activated macrophages enhance migratory potential of breast cancer cells which was supressed by IL-6 neutralization in CM of activated macrophages or inhibition of STAT-3 in breast cancer cells indicating the role of TAM derived IL-6/STAT3 pathway in metastasis of breast cancer. It has been reported that IL-6 can enhance tumor angiogenesis and metastasis either directly or by inducing the expression of well-known angiogenic and metastatic molecules such as VEGF, MMP2 and MMP9 in cancer cells [41–43]. Our findings from HUVEC tube formation studies depicted that TAM derived IL-6 promotes angiogenesis either directly or by inducing angiogenic potential of breast cancer cells via STAT-3 pathway. Earlier, Nagasaki et al. demonstrated that IL-6 regulates tumor angiogenesis using in vitro and in vivo models in colon cancer [42]. Further, Bharti et al. have shown that Il-6 induces angiogenesis under in vitro as well as in vivo conditions in breast cancer [44]. These studies support our results and indicate that TAM derived IL-6 may induce breast tumor angiogenesis under in vivo conditions.

Further, to validate our in vitro findings under in vivo conditions, we examined the effect of CM of activated macrophages on 4T1 derived tumor growth in BALB/c mice. The data showed that CM of activated macrophages enhanced tumor growth as compared to control whereas neutralization of IL-6 in CM of activated macrophages abrogated the effect. Thus, our data suggests that activated macrophages derived IL-6 have the ability to induce tumor growth by inducing cell proliferation which is evident by enhanced Ki67 expression in activated macrophage derived CM treated tumors. Furthermore, to examine whether the TAMs induced tumor growth is occurred through enrichment of CSCs, we establish the primary cultures from tumors and FACS analysis was performed to sort the cancer cells. It was demonstrated that CM of activated macrophage derived tumors have higher CSC population as compared to control.

**Fig. 7:**
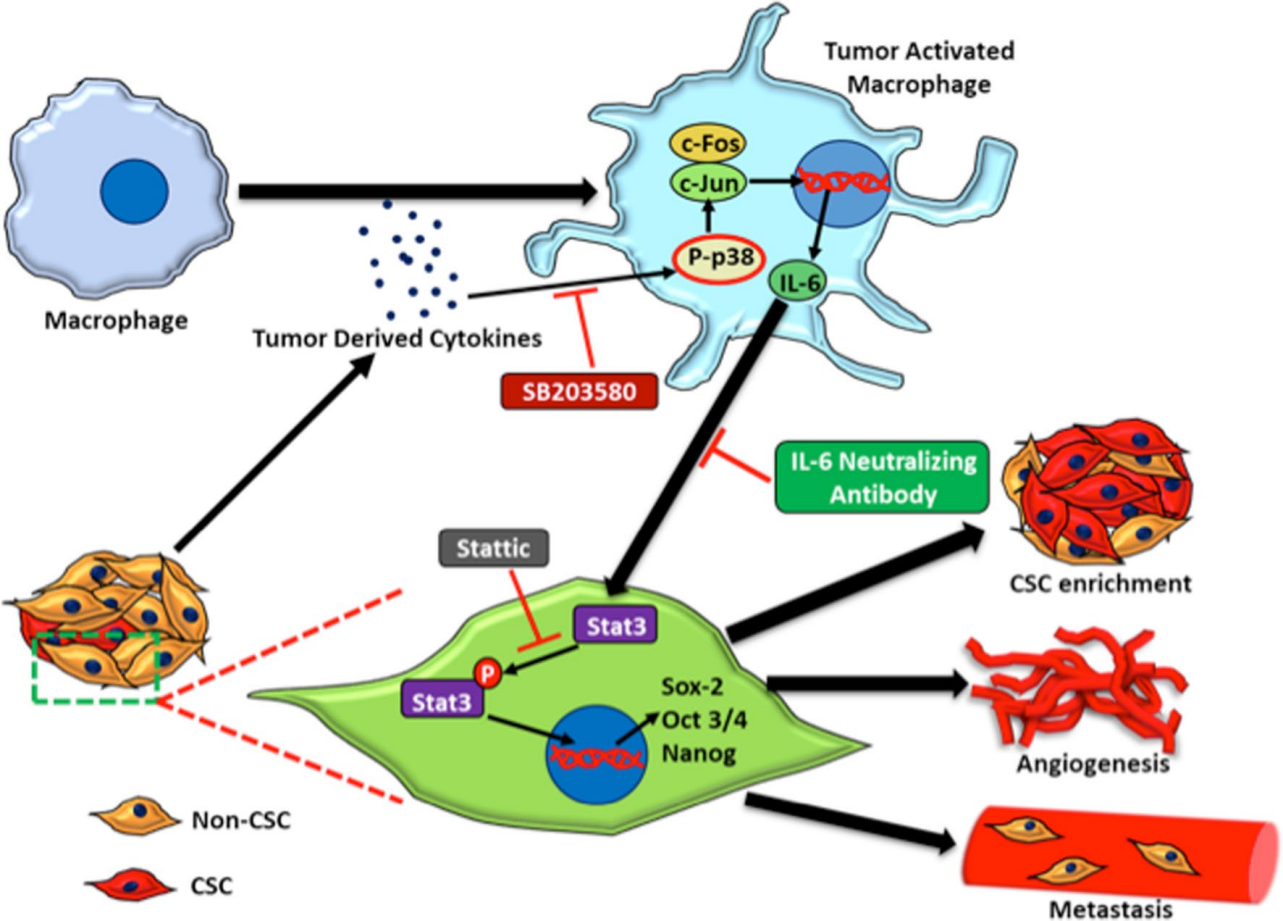
TAM derived IL-6 induces CSC enrichment and breast tumor growth in breast cancer. Proposed working model showing the breast cancer mediated activation of macrophages leads to enhanced IL-6 production. Further, activated macrophages derived IL-6 regulates breast cancer stem cell enrichment and subsequent tumor progression through STAT-3 pathway

We acknowledge certain limitations of our study including use of single breast cancer cell line. Since this study have been performed in murine model, use of human macrophages and breast cancer cells would be useful in clinical implementation of current study. Further, studies have shown that 4T1 cells are known to secrete more myeloid regulatory factors therefore including additional cells line would be useful for comparative study [45]. Further, expression of IL-6 receptor could have been examined in breast cancer cells for more conclusive demonstration of TAM derived IL-6 mediated activation of STAT-3 pathway in breast cancer cells. However, Oh et al. have demonstrated that myeloid-derived suppressor cells derived IL-6 induced metastasis in murine breast cancer cells by activating STAT3 pathway via soluble IL-6Rα mediated trans-signaling [12]. Similar mechanism may be involved in our study however further experiments are required to validate this hypothesis. Nevertheless, here we have proved by various methods that breast cancer cells can activate macrophages into TAMs and these TAM derived IL-6 induces CSC enrichment and tumor progression in breast cancer (Fig. 7).

## Conclusion

In conclusion, we herein provide direct evidence that breast cancer cells educate macrophages towards tumor promoting phenotype by inducing IL-6 expression through p38-MAPK pathway. Our results depict that activated macrophages derived IL-6 regulates CSC enrichment, tumor growth, metastasis and angiogenesis in breast cancer through STAT-3 pathway. These data suggested that blocked of crosstalk between TAMs and cancer cells may redirect the macrophage function and anti-IL-6 therapy combined with inhibition of STAT-3 signaling may be a useful approach for targeting tumor growth and angiogenesis.

## Supplementary Information

**Additional file 1.** **Figure S1**. Murine macrophages (RAW264.7) were treated with CM of breast cancer cells (4T1) for 24 h and change in morphology was examined by phase contrast microscope. **Figure S2**. (**A**-**C**) Dot-plots from TCGA data for breast cancer from PanCancer Atlas, METABRIC and The Metastatic Breast Cancer Project (MBC) datasets to illustrate correlations between CD163 and IL-6 expression. **Figure S3**. Effect of CM of breast cancer cells on IL-6 expression in activated macrophages was examined by (**A**, **B**) confocal microscopy (**C**) ELISA and (**D**) FACS analysis was performed for IL-6 expression using FACS Canto II analyser.** Figure S4**. Tumor activated macrophages promote stem cell phenotype in breast cancer as determined by examining of expression of (**A**) Sca-1 as CSC marker by FACS and (**B**-**G**) Sox-2, Oct 3/4 and Nanog by q-PCR and immunofluorescence.** Figure S5**. Comparison of IL-6 level in 4T1 and Activated RAW conditioned media. Bar graph represents IL-6 concentration in CM of 4T1 cells and CM of Activated RAW cells estimated by ELISA.** Figure S6**. TAM derived IL-6 enriches CSCs in breast cancer. 4T1 cells were treated with either CM of activated RAW or CM of activated RAW neutralized with IL-6 antibody (20 μg/ml) and co-expression ALDH1 as well as Sca-1 was examined by flow cytometry using their specific antibody.** Figure S7**. IL-6 alone is sufficient to enhance cancer stem cell phenotype in breast cancer cells as shown by (**A**,** B**) flow cytometry (**C**) western blotting and (**D**-**F**) confocal microscopy.** Figure S8**. IL-6 enhances stemness in breast cancer through STAT-3 pathways demonstrated by flow cytometry.** Figure S9**. TAM derived IL-6 augments migration in breast cancer cells as determined by wound healing assay.** Figure S10**. Tumor activated macrophages enrich CSC population in orthotopic breast cancer model. Digital photographs of tumor bearing BALB/c.** Table S1**: List of Antibodies, kits, inhibitors and recombinant proteins used.** Table S2**: List of Primers Used in study.

## Data Availability

All the data generated or analyzed during this study are included in this article and its additional files.
