# Supplementary material for: Tumor-associated macrophage derived IL-6 enriches cancer stem cell population and promotes breast tumor progression via Stat-3 pathway
Source: Cancer Cell Int. 2022 Mar 17;22:122. doi: 10.1186/s12935-022-02527-9 (PMC8932105; doi:10.1186/s12935-022-02527-9)
Supplement: Supplementary file 1 — Additional file 1. Figure S1. Murine macrophages (RAW264.7) were treated with CM of breast cancer cells (4T1) for 24 h and change in morphology was examined by phase contrast microscope. Figure S2. (A-C) Dot-plots from TCGA data for breast cancer from PanCancer Atlas, METABRIC and The Metastatic Breast Cancer Project (MBC) datasets to illustrate correlations between CD163 and IL-6 expression. Figure S3. Effect of CM of breast cancer cells on IL-6 expression in activated macrophages was examined by (A, B) confocal microscopy (C) ELISA and (D) FACS analysis was performed for IL-6 expression using FACS Canto II analyser. Figure S4. Tumor activated macrophages promote stem cell phenotype in breast cancer as determined by examining of expression of (A) Sca-1 as CSC marker by FACS and (B-G) Sox-2, Oct 3/4 and Nanog by q-PCR and immunofluorescence. Figure S5. Comparison of IL-6 level in 4T1 and Activated RAW conditioned media. Bar graph represents IL-6 concentration in CM of 4T1 cells and CM of Activated RAW cells estimated by ELISA. Figure S6. TAM derived IL-6 enriches CSCs in breast cancer. 4T1 cells were treated with either CM of activated RAW or CM of activated RAW neutralized with IL-6 antibody (20 μg/ml) and co-expression ALDH1 as well as Sca-1 was examined by flow cytometry using their specific antibody. Figure S7. IL-6 alone is sufficient to enhance cancer stem cell phenotype in breast cancer cells as shown by (A, B) flow cytometry (C) western blotting and (D-F) confocal microscopy. Figure S8. IL-6 enhances stemness in breast cancer through STAT-3 pathways demonstrated by flow cytometry. Figure S9. TAM derived IL-6 augments migration in breast cancer cells as determined by wound healing assay. Figure S10. Tumor activated macrophages enrich CSC population in orthotopic breast cancer model. Digital photographs of tumor bearing BALB/c. Table S1: List of Antibodies, kits, inhibitors and recombinant proteins used. Table S2: List of Primers Used in study. [file 12935_2022_2527_MOESM1_ESM.pdf]

Tumor-associated macrophage derived IL-6 enriches cancer stem cell population and promotes tumor progression in breast cancer via Stat-3 pathway

NNV Radharani* [1], Amit S. Yadav* [1,2], Ramakrishna Nimma[1], TV Santosh Kumar[1], Anuradha Bulbule[1], Venkatesh Chanukuppa[5], Dhiraj Kumar[4], Srinivas Patnaik[2], Srikanth Rapole[5], Gopal C. Kundu# [1,2,3]

[1]Laboratory of Tumor Biology, Angiogenesis and Nanomedicine Research, National Centre for Cell Science (NCCS), Pune 411007, India.

[2]School of Biotechnology and [3]Kalinga Institute of Medical Sciences (KIMS), KIIT Deemed to be University, Bhubaneswar 751024, India.

[4]Present Address: Department of Cancer Biology, The University of Texas MD Anderson Cancer Center, Houston, Texas, 77054, USA

[5]Proteomics Laboratory, National Centre for Cell Science (NCCS), Pune 411007, India

#Correspondence: School of Biotechnology, KIIT Deemed to be University, Bhubaneswar 751 024, India.

Email: gopalc.kundu@kiit.ac.in

*These authors contributed equally to this work

Supplementary Methods

Secretomic analysis

To examine the status of cytokine profile in macrophages in response to 4T1 cells, quantitative secretomics was performed using label free conditions. In brief, RAW264.7 cells were either cultured alone or treated with CM of 4T1 for 12 h and conditioned media were collected. The concentrated conditioned media were further replaced using 0.5 M Tri ethyl ammonium bicarbonate (TEAB) by performing centrifugation at 16000 rpm at 15 °C. Further, secretome protein estimation was performed using 2D quantification kit (Thermofisher) and label free quantification was performed for secretome analysis.

Fifty micro gram protein was used either from control or treated sample for enzymatic protein digestion. Briefly, control and treatment samples were first reduced using 10 mM DTT, followed by 50 mM Iodoacetamide (IAA) alkylation and samples were digested by trypsin (1:50; enzyme: protein). Further the peptides were desalted using zip-tips and suspended in 0.1% formic acid (Sigma Aldrich, USA) containing LC-MS grade water (JT Baker, USA). For LFQ data acquisition, Orbitrap fusion mass spectrometer (ThermoScientific™, USA) coupled with EASY-nLC 1200 nanoflow liquid chromatography system (ThermoScientific™, USA) equipped with EASY- Spray column (50 cm × 75 µm ID, PepMap C18) was used. One microgram of desalted tryptic peptide samples with three biological replicates were used from each sample and injected to Orbitrap Fusion mass spectrometer. Further, peptides were separated using 5 to 95% phase B (0.1% formic acid in 80% acetonitrile) and 300 nL/min flow rate for 140 min through gradient process. MS scan analysis was performed by analysing MS1 spectrum within 375-1500 m/z mass range at 60,000 resolution using Orbitrap analyser with 50 ms injection time. Further the identified MS1 precursors had undergone fragmentation by high energy collision-induced dissociation (HCD) and analysed by Orbitrap mass analyser.

Further proteins were identified and quantified from the mass spectrometry data using proteome Discoverer software (PD) (version 2.2, Thermo Scientific) by using Sequest HT database. Database search includes all entries from the Mus musculus UniProt reference proteome database. Fold change values were obtained based on the mean area from all triplicate samples.

Isolation of TAMs from 4T1 breast tumors

To isolate TAMs from tumors, 4T1 cells ($2x10^5$) were implanted into the mammary fat pads of female BALB/c mice and tumors were allowed to grow up to standard size. Mice were sacrificed and tumors were dissected and collected. Briefly, tumors were chopped and treated with collagenase IV and DNAse I for 45 mins. Cells were passed through 100 mm strainer and centrifuged at 2000 rpm for 5 mins at 4 °C. Supernatant was removed and pellet was suspended in 1 ml of staining buffer and anti-F4/80 antibody (1:100) was added and incubated in ice for 45 mins in dark along with their respective matched unstained control. Cell were then washed twice with sterile 1X PBS. The stained cells were sorted on FACSAria III or FACS Aria II (BD-Biosciences). CD11b positive cells were collected in collection tubes for further experiments.

Flow cytometry

For FACS analysis, RAW264.7 cells were treated with either CM of 4T1 or pre-treated with 10 µM of SB203580 for 1 h, followed by treatment with CM of 4T1 for 24 h and further treated with Brefaldin for 4 h and levels of expression of IL-6 were analysed. For intracellular staining of IL-6, RAW264.7 cells were scraped after treatments, suspended in 1X PBS and centrifuged at 1000 rpm for 10 mins. Cells were fixed using 2% Formaldehyde for 20 mins in ice followed by permeabilization with chilled methanol for 30 mins at 4 °C. Cells were washed with 1X PBS until methanol was completely removed followed by suspension in specific antibody. In separate studies, RAW264.7 cells were co-cultured with CM of 4T1 for 24 h and CD206 expression was analysed. In brief, cells were trypsinized and re-suspended in 100 µl

of 1X PBS containing 3% FBS and anti-CD206 or anti-IL-6 antibody was added, incubated for 45 mins on ice in dark along with their respective matched unstained control and then washed twice with 1X PBS. Stained cells were analysed on FACSCanto (BD-Biosciences) and data was analysed using FACS DIVA software (Version 6.1.3).

To study the role of TAM derived IL-6 in enrichment of CSCs through STAT-3 pathway in breast cancer cells, 4T1 cells were treated with either CM of activated macrophages or IL-6 neutralized CM of activated macrophages or pre-treated with Stattic for 3 h followed by treatment with CM of activated macrophages for 24 h and processed for FACS analysis. In brief, cells were trypsinized and re-suspended in 100 µl of 1X PBS containing 3% FBS and anti-Sca-1 antibody was added, incubated for 45 mins along with their respective matched unstained control and then washed twice with 1X PBS. Stained cells were examined on FACSCanto (BD-Biosciences) and data was analysed using FACS DIVA software (Version 6.1.3).

Chromatin immunoprecipitation (ChIP) assay

The IL-6 promoter sequence and putative AP-1 binding site was obtained from The Eukaryotic Promoter Database (EPD) and primers were designed for binding site (Table S2). The untreated or 4T1 CM-treated RAW264.7 cells were fixed with 0.75% formaldehyde for 10 min followed by quenching with 125 mM glycine for 5 min at RT. After centrifugation, cell pellets were resuspended in FA lysis buffer followed by sonication. 10% lysate removed for Input. Primary antibody (anti-cJun antibody) was used for immunoprecipitation at 4°C. The immuno-precipitate was then pulled down with SureBeads Protein G magnetic beads and eluted with elution buffer. The immuno-precipitates were then reverse cross-linked using RNase A and Proteinase K and purified. Semi-quantitative PCR was used to quantify DNA levels of specific loci by analyzing PCR end-product using agarose gel electrophoresis.

Aldefluor assay

4T1 single-cell suspensions were used for Aldefluor assay (Stem Cell Technologies) as per the manufacturer's directions. In brief, 4T1 cells ($1x10^{6)}$ were re-suspended in 1 ml Aldefluor buffer containing activated Aldefluor substrate. As a negative control, for each sample ALDH inhibitor, DEAB (diethylaminobenzaldehyde) was used. Stained cells were examined on FACSCanto (BD-Biosciences) and data was analysed using FACS DIVA software (Version 6.1.3).

Transwell migration assay:

4T1 cell ($1 \times 10^{4)}$ either untreated or treated with CM of Act RAW264.7 were seeded in the upper chamber. In separate experiments, 4T1 cells were treated with IL-6 neutralized CM of activated macrophages or pre-treated with Stattic for 3 h followed by CM of activated macrophages seeded in the upper chamber. 10% FBS was used in lower chambers as a chemoattractant. The migrated cells were stained with crystal violet, counted in five high-power fields (hpfs) and analysed.

Wound healing assay

The wound healing assay was performed using 4T1 cells under different experimental conditions. Briefly, 4T1 cells were grown in monolayer until formed cobblestone morphology. Uniform sized wounds were made using sterile tip and cells were co-cultured with either CM of activated macrophages or IL-6 neutralized CM of activated macrophages or 4T1 cells pre-treated with Stattic for 3 h followed by CM of activated macrophages. Untreated 4T1 cells were used as control. Photographs of wounds were taken at t = 0 h and t = 18 h using phase contrast microscope (Nikon). Area migrated was analysed by using Image-Pro plus software and displayed in the form of bar graph.

Supplementary Figures

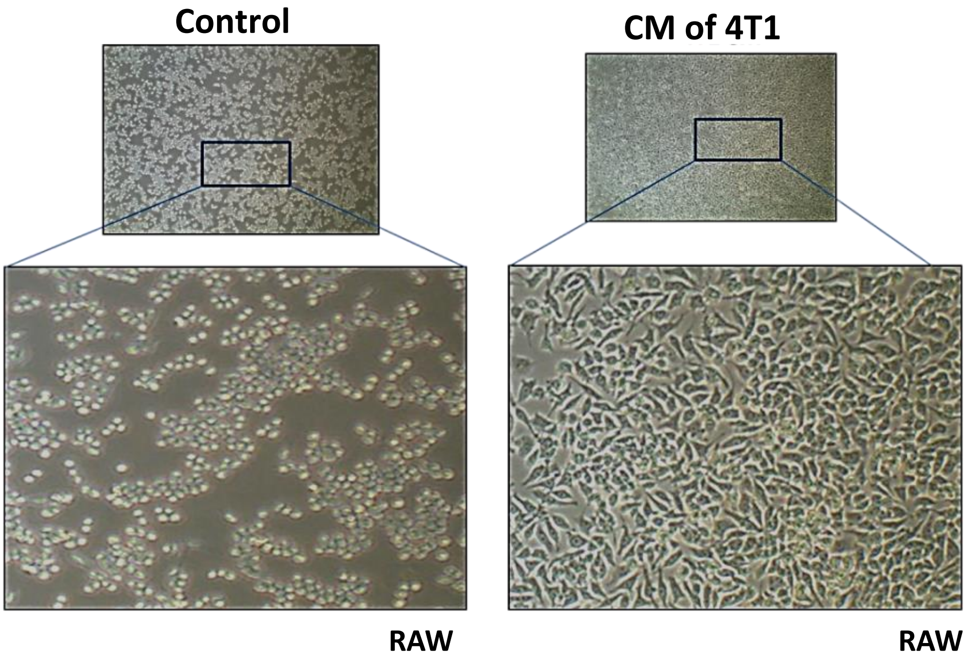

Figure S1. Breast Cancer Conditioned Media (CM) activates macrophages. (A) Murine macrophages (RAW264.7) were treated with CM of breast cancer cells (4T1) for 24 h and change in morphology was examined by phase contrast microscope.

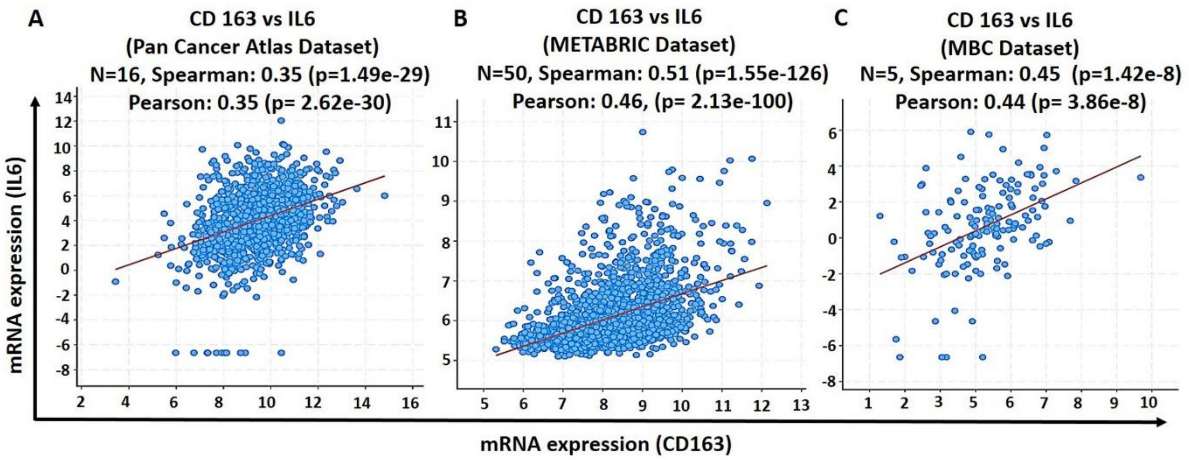

Figure S2. IL-6 expression is positively correlated with TAM specific marker expression in breast cancer clinical specimen. (A-C) Dot-plots from TCGA data for breast cancer from PanCancer Atlas, METABRIC and The Metastatic Breast Cancer Project (MBC) datasets to illustrate correlations between CD163 and IL-6 expression.

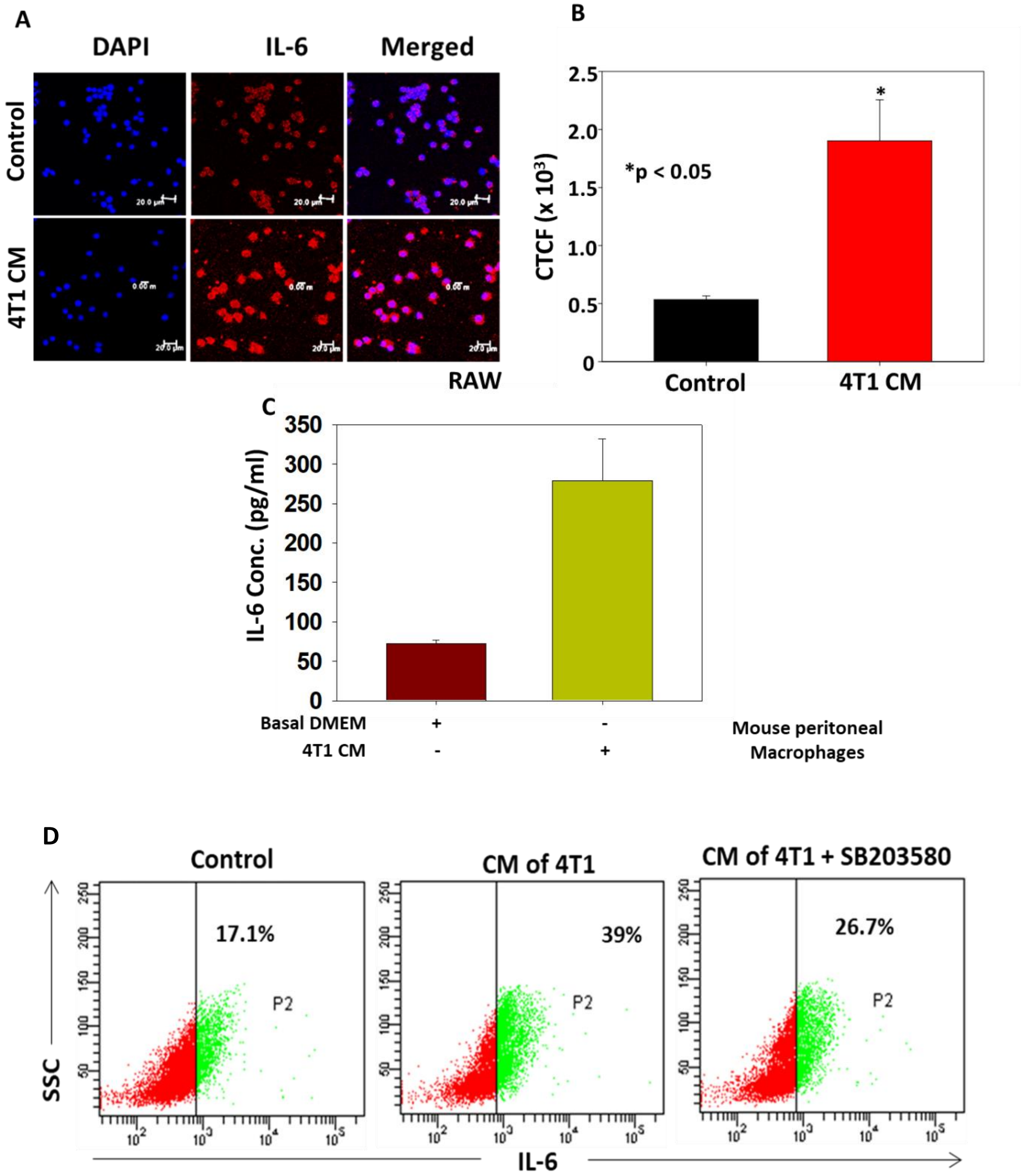

Figure S3. CM of breast cancer cells promote IL-6 expression in activated macrophages. (A) RAW264.7 cells were treated with CM of 4T1 cells for 24 h. Brefeldin A was added to inhibit the secretion of IL-6. After treatment, cells were processed and analysed for IL-6 expression using confocal microscopy. DAPI was used to stain the nuclei. **(B)** Bar graph represents Corrected Total Cell Fluorescence (CTCF) from three independent biological experiments (n= 3). Error bars represent mean ± SEM, *denotes $p < 0.05$. **(C)** Bar graph represents IL-6 concentration in CM of activated RAW264.7 cells vs control as examined by ELISA. Error bars represent mean ± SEM, n=2 independent experiments. **(D)** RAW264.7 cells were either treated with 4T1 CM alone or pre-treated with SB203580 followed by treatment with 4T1 CM and FACS analysis was performed for IL-6 expression using FACS Canto II analyser

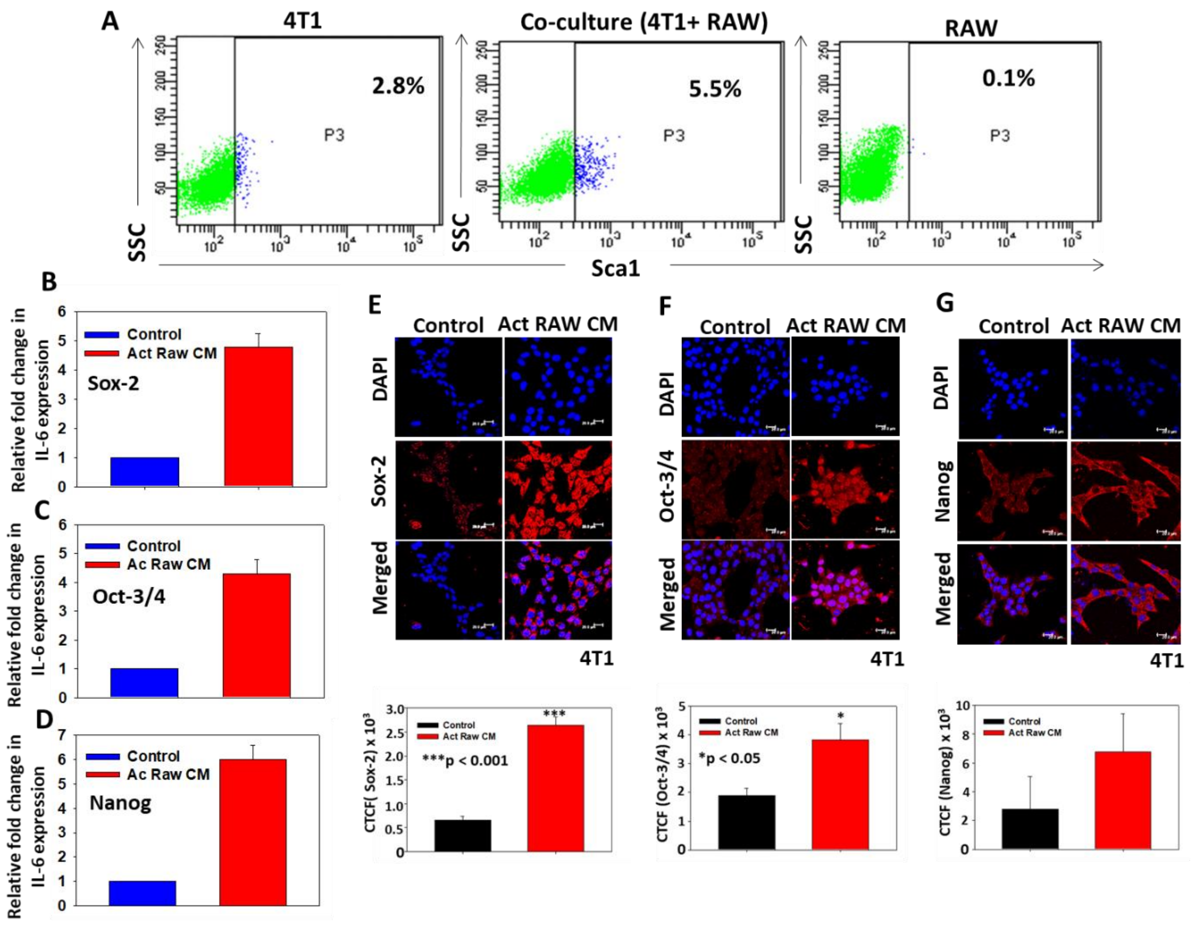

Figure S4. Tumor activated macrophages promote stem cell phenotype in breast cancer. (A) 4T1 cells either cultured alone or co-cultured with RAW264.7 macrophages for 24 h. Enrichment of breast CSCs was analysed by FACS using Sca-1 as CSC marker. **(B-D)** 4T1 cells were treated with CM of activated RAW264.7 cells for 24 h and expressions of Sox-2, Oct 3/4 and Nanog were analysed by q-PCR. Bar graphs represent relative fold change in IL-6 expression of Sox-2, Oct-3/4 and Nanog respectively. **(E-G)** 4T1 cells were treated with CM of activated RAW264.7 cells for 24 h and expressions of Sox-2, Oct 3/4 and Nanog were analysed by confocal microscopy. Bar graphs represent corrected total cell fluorescence (CTCF) of Sox-2, Oct-3/4 and Nanog respectively as quantified by ImageJ software, mean ± SEM, *denotes $p < 0.05$, ***denotes $p < 0.001$, n=3 independent experiments.

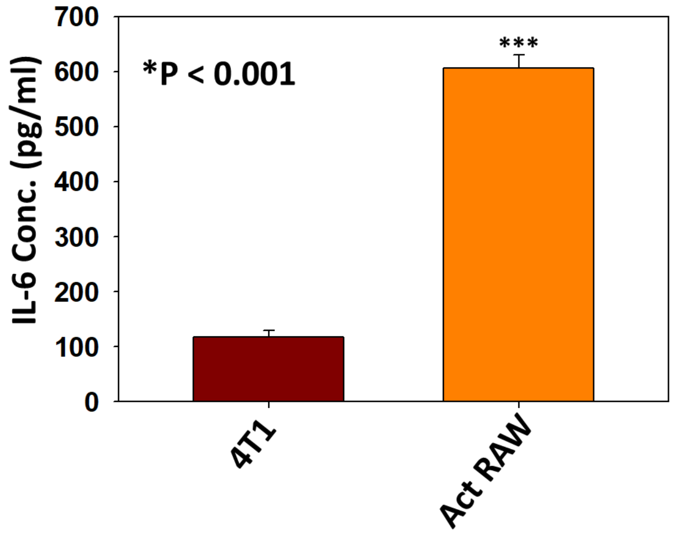

Figure S5. Comparison of IL-6 level in 4T1 and Activated RAW conditioned media. Bar graph represents IL-6 concentration in CM of 4T1 cells and CM of Activated RAW cells estimated by ELISA, mean ± SEM, ***denotes $p < 0.001$, n=3 independent experiments.

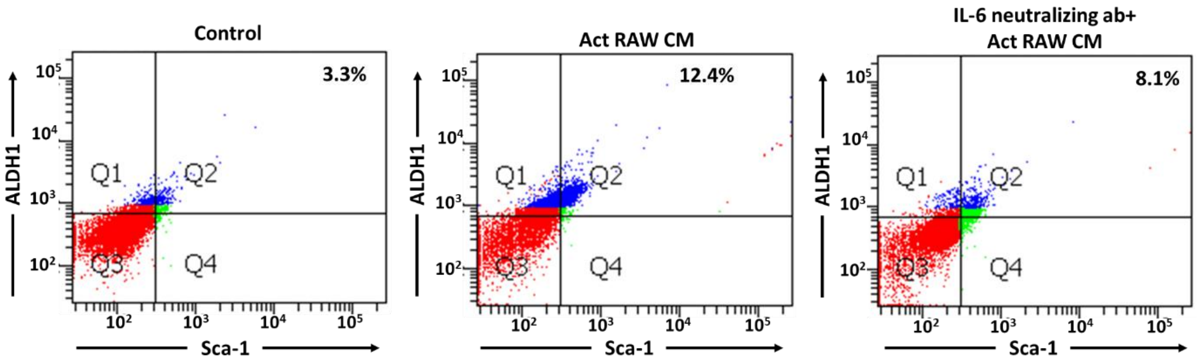

Figure S6. TAM derived IL-6 enriches CSCs in breast cancer. 4T1 cells were treated with either CM of activated RAW or CM of activated RAW neutralized with IL-6 antibody (20 µg/ml) and co-expression ALDH1 as well as Sca-1 was examined by flow cytometry using their specific antibody.

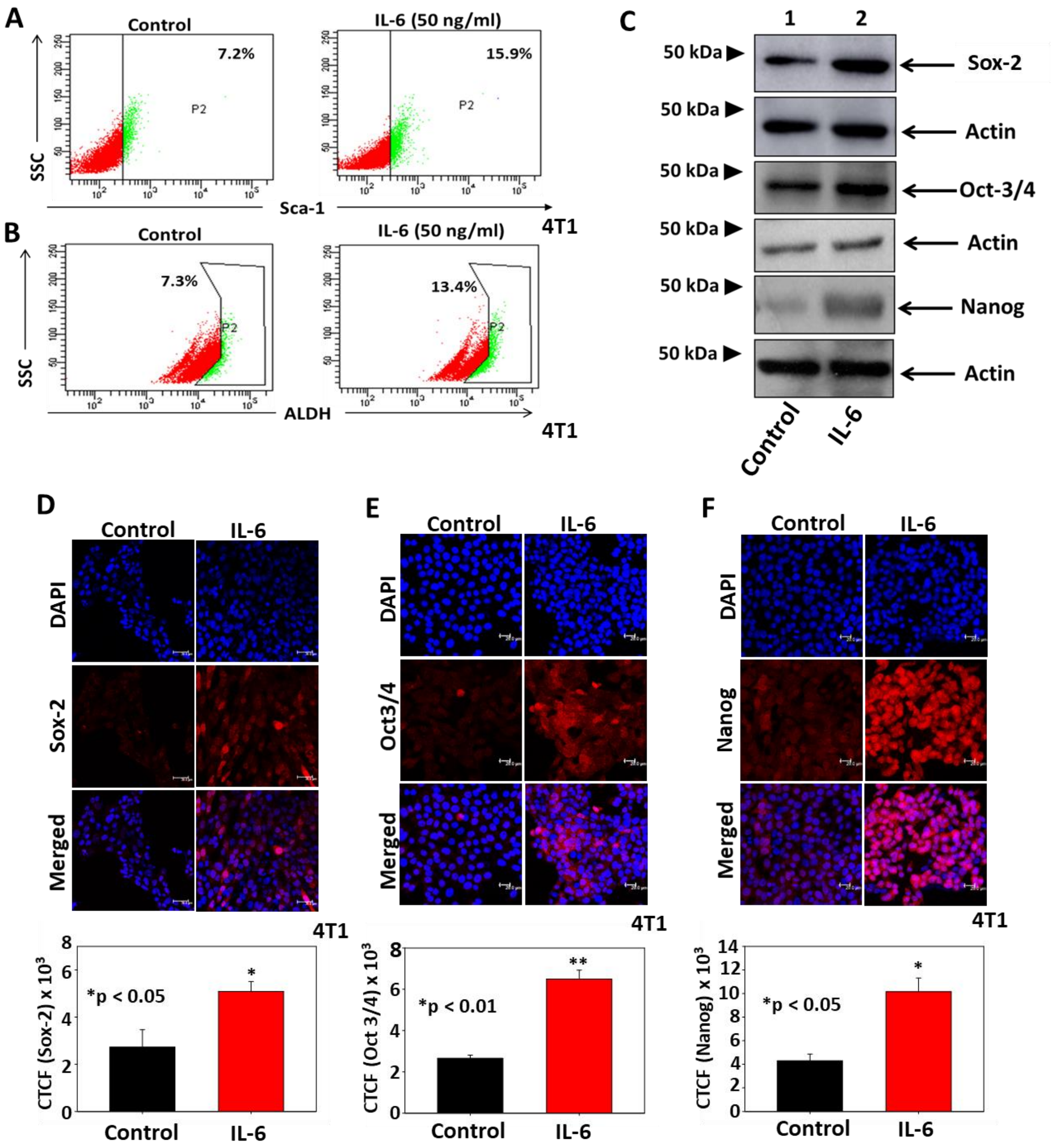

Figure S7. IL-6 alone is sufficient to enhance cancer stem cell phenotype in breast cancer cells. (A, B) 4T1 cells were treated with recombinant IL-6 (50 ng/ml) for 24 h and CSC phenotype was examined by analysing Sca-1 expression and ALDH1 activity using flow cytometry. **(C)** 4T1 cells were treated with recombinant mouse IL-6 (50 ng/ml) for 24 h and expressions of Sox-2, Oct 3/4 and Nanog were analysed by western blot. **(D-F)** 4T1 cells were treated with recombinant mouse IL-6 (50 ng/ml) for 24 h and expressions of Sox-2, Oct 3/4 and Nanog were analysed by confocal microscopy. Bar graphs represent corrected total cell fluorescence (CTCF) of Nanog, Oct-3/4 and Sox-2 respectively as quantified by ImageJ software, mean ± SEM, *denotes $p < 0.05$, **denotes $p < 0.01$, n=3 independent experiments).

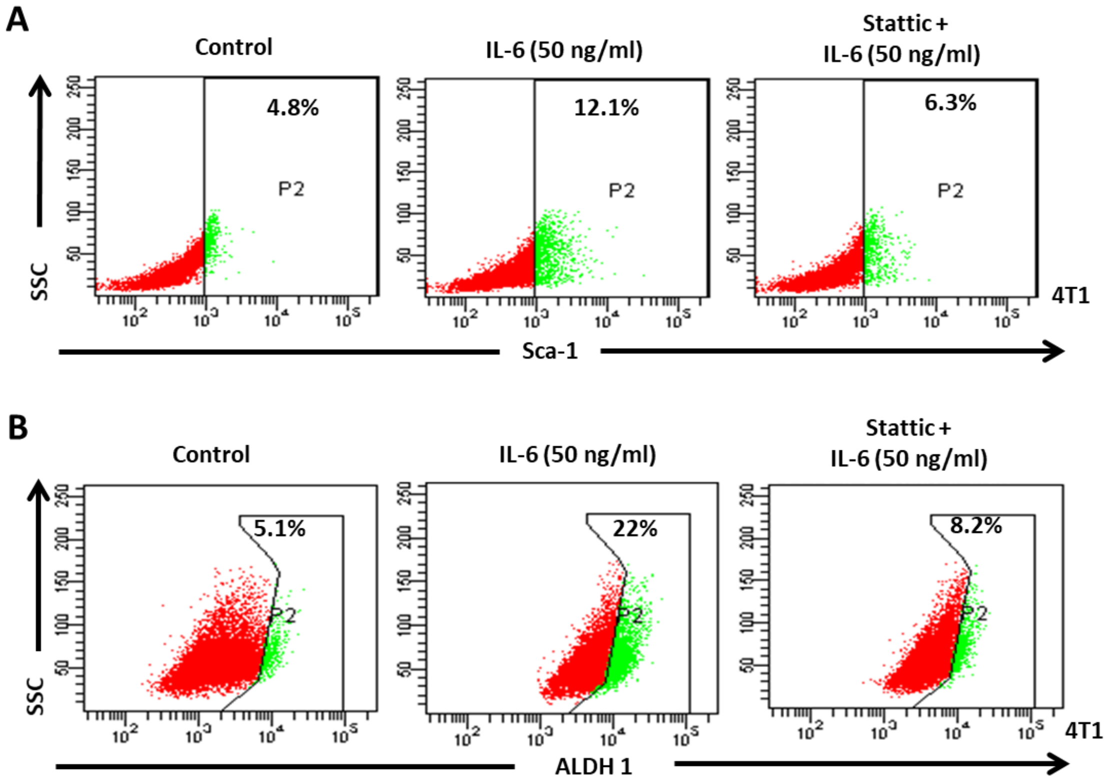

Figure S8. IL-6 enhances stemness in breast cancer through STAT-3 pathways. (A, B) 4T1 cells were treated with Stattic (5 μM), a STAT-3 inhibitor, prior to the treatment with recombinant IL-6 and CSC phenotype was examined by analysing Sca-1 expression and Aldefluor activity using flow cytometry.

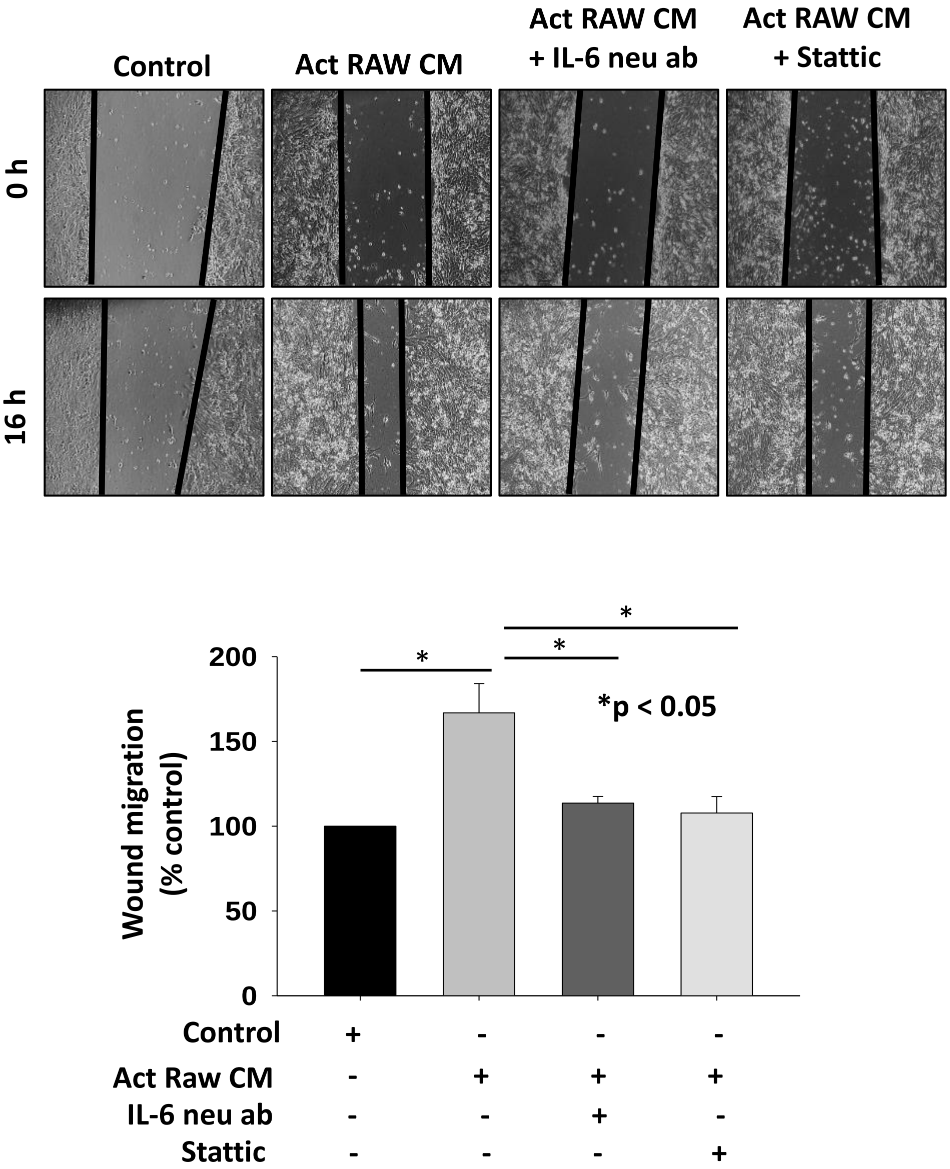

Figure S9. TAM derived IL-6 augments migration in breast cancer cells. 4T1 cells were grown in confluent monolayer with cobblestone morphology and wounds with constant width were given. Cells were treated with CM of activated RAW264.7 cells or CM neutralised with IL-6 antibody (20 µg/ml). In separate experiments, 4T1 cells were pre-treated with Stattic (5 µM) followed by treatment with CM of activated RAW264.7 cells. Wounds were imaged at 0 h and 16 h and area migrated was analysed using Image-Pro Plus software and represented in the form of bar graph. The error bar represents mean ± SEM, *denotes $p < 0.05$, n=3 independent experiments.

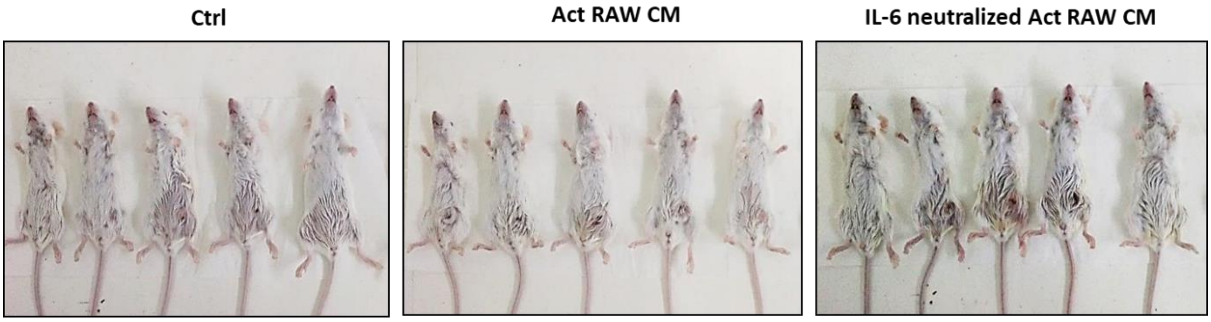

Figure S10. Tumor activated macrophages enrich CSC population in orthotopic breast cancer model. Digital photographs of tumor bearing BALB/c

Table S1: List of Antibodies, kits, inhibitors and recombinant proteins used

Sr No.	Product	Company	Cat No	Origin	Use
1	PE anti-mouse Sca-1	Biolegend	108120	Rat	FACS
2	Anti-mouse IL-6	Biolegend	504512	Rat	Neutralization
3	PE anti-mouse IL-6	Biolegend	504504	Rat	FACS
4	APC anti-mouse CD206	Biolegend	141708	Rat	FACS
5	Anti-IL-6 Ab	Santacruz	sc-1265	Goat	Immunofluorescence
6	Nanog	Santacruz	sc-30328	Goat	Immunofluorescence, Western blot
7	Sox-2	Santacruz	sc-365823	Mouse	Immunofluorescence, Western blot
8	Oct-3/4	Santacruz	sc-5279	Mouse	Immunofluorescence, Western blot
9	Actin	Santacruz	sc-1615	Goat	Western blot
10	c-Fos	Santacruz	sc-52	Rabbit	Immunofluorescence
11	c-Fos	Cell Signaling Technology	2250S	Rabbit	Western blot
12	c-Jun	Santacruz	sc-45	Rabbit	Immunofluorescence
13	c-Jun	Cell Signaling Technology	9165S	Rabbit	Western blot, ChIP
14	p-Stat3	Cell Signaling Technology	9145S	Rabbit	Immunofluorescence, Western blot
15	Stat3	Cell Signaling Technology	9139S	Mouse	Immunofluorescence, Western blot
16	Phospho-p38	Cell Signaling Technology	4511S	Rabbit	Immunofluorescence, Western blot
17	p38	Santacruz	sc-81621	Mouse	Western blot
18	Goat anti-rabbit IgG – HRP	GeNei	HPO3	Goat	Western blot
19	Rabbit anti-goat IgG – HRP	GeNei	HPO4	Rabbit	Western blot
20	Rabbit anti-mouse IgG – HRP	GeNei	HPO5	Rabbit	Western blot
21	ALDEFLUOR™ Kit	Stem cell technologies	1700	NA	FACS
22	Mouse IL-6 ELISA Kit	Biolegend	431301	NA	ELISA
23	Recombinant Mouse IL-6	Biolegend	575704	NA	Various experiments
24	Stattic	Sigma-Aldrich	S7947	NA	Various experiments (Inhibitor)
25	SB 203580	Sigma-Aldrich (Calbiochem)	559389	NA	Various experiments (Inhibitor)

Table S2: List of Primers Used in study

Gene	Forward	Reverse
	**q-PCR**	
**IL-6**	GAGGATACCACTCCCAACAGACC	AAGTGCATCATCGTTGTTCATACA
**β actin**	ATCAAGATCATTGCTCCTCC	AAAGAAAGGGTGTAAAACGC
	**ChIP**	
**AP-1**	CAGTAGAAGGGAGCTTCAAACAC	ACATTTCCAGGTCATGCAAGG
